# Temporal binding and social perception – A replication study

**DOI:** 10.1371/journal.pone.0352510

**Published:** 2026-07-14

**Authors:** David H.V. Vogel, Manuela Woschei, Mathis Jording, Peter H. Weiss, Kai Vogeley

**Affiliations:** 1 Department of Psychiatry and Psychotherapy, University Hospital Bonn, Bonn, Germany; 2 Department of Psychiatry, Faculty of Medicine and University Hospital Cologne, University of Cologne, Cologne, Germany; 3 Department of Neurology, Faculty of Medicine and University Hospital Cologne, University of Cologne, Cologne, Germany; 4 Research Center Juelich, Institute of Neuroscience and Medicine, Cognitive Neuroscience (INM3), Juelich, Germany; University of Bologna, ITALY

## Abstract

Previous studies reported action-effect binding for social contexts in a variety of experimental contexts. When confronted with social information, perceptual or conceptual, participants underestimated the duration of time intervals between their actions and subsequent social events, as compared to only observing social events. Current evidence demonstrates stronger temporal binding both for conditions of believing in an interaction with a social partner, and for perceiving an interaction partner. Fewer data exist about the influence of exclusively perceptual social cues such as interaction with a face stimulus. We here report on a study investigating this so-called social hyperbinding for face perception. 40 participants performed a duration estimation task in four blocks involving either the active manipulation of gaze direction, the active manipulation of a geometric pattern, the passive observation of gaze direction, or the passive observation of movement in a geometric pattern. Although results indicated trends towards shorter time estimates for the active manipulation of gaze, with no visible differences in time perception for the other three conditions, our analysis did neither confirm temporal binding nor social hyperbinding for face stimuli. This finding challenges the theory of social hyperbinding for face stimuli. While stronger temporal binding for real and believed interactions with another human has been reliably replicated in the literature, the results from this experiment cannot confirm increased binding for face-like stimuli.

## 1. Introduction

Repeated investigation has demonstrated an influence of socio-cognitive processes on time perception [1–4]. More recently, paradigms investigating temporal binding (TB) during social interaction revealed a distinct influence of social factors [5–12]. TB refers to the relative underestimation of time intervals when the respective duration involves a preceding voluntary action [13,14]. Estimates by participants of time intervals between a voluntary action and a subsequent event are systematically shorter than estimates of time intervals between two arbitrary events of the same duration [14,15].

Studies involving social action and interaction repeatedly demonstrated an increase of TB for social event sequences. Most interestingly, this social hyperbinding [9] has been successfully replicated for event sequences involving the belief in another person [10]. In other words, it appears that both social stimuli (“bottom-up”) and social beliefs (“top-down”) are sufficient to elicit social hyperbinding.

During a simple TB experiment, participants judge durations between for example, key presses and subsequent auditory stimuli [15]. These ratings are compared to judgments of durations of identical lengths between for instance, two auditory stimuli. Across participants, the former judgements turn out shorter than the latter judgements. However, while watching another person perform the task [6] or when performing the task with another person [5,8,16], TB is smaller than while performing the task alone. A similar decrease in TB occurs when being ordered to perform actions as compared to performing actions at one’s own accord, while TB is stronger when leading actions of others [7].

The influence of a social context further becomes evident when actions result in changes in social stimuli. For example, when participants caused eye movements in a realistic picture of a face, durations judgments where shorter than when causing changes in scrambles of these pictures [17]. Ulloa et al. [18] relatedly demonstrated shorter time estimates when judging eye movements in realistic face stimuli.

These previous findings hold various implications, as, in theory, TB relates to different cognitive processes. For one, TB has been theorized to depend on the Sense of Agency – the feeling of being in control of one’s actions and their consequences (for review see [13,14]). It has been argued, that social hyperbinding relates to an increase in agency by the induction of joint agency [5,6]. The increase in control brought on by the interaction appears to be associated with the temporal compression between action and event.

The Sense of Agency account of temporal binding has, however, increasingly come under scrutiny over the past years. The critique is brought on by a surging number of findings indicating TB without intentional action [19–21]. Subsequent theory postulates that TB corresponds to causality and predictability [22–25]. The more information available about any succession of events, the more predictable the sequence becomes. With increasing predictability and temporal contiguity neural predictive processing improves. This improvement in turn results in the temporal compression detectable as TB [25–27].

Intentional action – action involving a Sense of Agency – poses a special subtype of such cause-effect sequences. During voluntary actions and their outcomes, the available information is maximized, as the sequence does not only involve bottom-up perceptual information, but also extensive top-down information in the form of believes and assumptions available prior to and during the performance of the action-event sequence [22,24,25].

**Fig 1:**
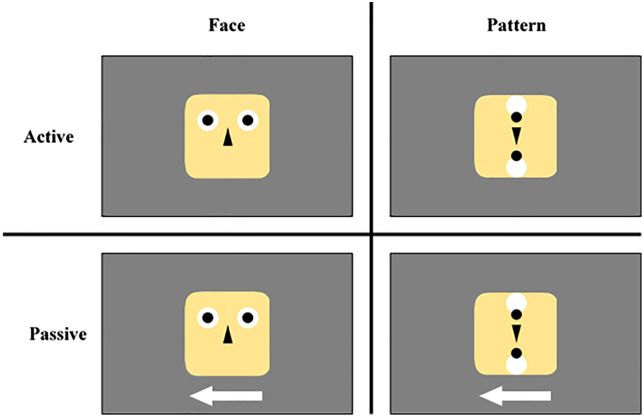
Block design for the experiment based on Vogel et al. (2021,2022). Participants performed 60 trials in four blocks. For active blocks (top row), participants pressed a key to cause either eye movements in a face (left column) or movements of dots in a geometric pattern (right column). For passive blocks participants observed the identical movements occurring after the appearance of an arrow below the stimulus. This resulted in the four combinations of active/face, active/pattern, passive/face, and passive/pattern.

Here, we wish to complement the two earlier studies by Vogel et al. [9,10]. In their studies, the authors completed a total of four experiments reporting social hyperbinding. In either study, the authors performed experiments designed to elicit a maximum effect of social influences on TB. In all experiments, participants judged time intervals between a left or right key press and a subsequent left or right movement on their computer screen. These intervals were compared to judgements of durations between the appearance of an arrow indicating either left or right and a subsequent left or right movement on the computer screen. The movement was either an eye movement of a face stimulus, or a movement in a geometric field composed of the same patterns as the face stimulus (see Fig 1). To maximize the available social information, participants were introduced to a confederate prior to the experiment and instructed that for all experimental blocks during which they were to see a face stimulus, the eye movements of the stimulus represented the eye movements of the confederate seated in front of an eye tracker in an adjacent room, responding to either theirs, or a computer’s commands. In reality, participants were always performing the task in interaction with a fully controlled experimental setup based on a computer algorithm.

Both studies report shorter time intervals for actions during social conditions. Whenever participants caused the eyes of a face to move while simultaneously thinking that they were interacting with another person, TB was stronger.

A second experiment reported in Vogel et al. (2022) [10] focused on the influence of social belief on TB. As in the experiment described above, the paradigm compared time judgements of intervals between a key press and a subsequent movement in a geometric field with judgments of intervals between the appearance of an arrow key and a subsequent movement. For half of the trials participants were made to believe they were interacting with another person, while during the other half, they were told they would be performing the experiment by themselves. Participants were, however, always performing the experiment by themselves. Just as in the previous experiment, judgements for durations between actions and movements were significantly shorter when participants believed to be interacting with a partner.

Vogel et al. (2021) [9] complement this finding by manipulating the combination of top-down social belief – meaning the belief in the confederate as an interactant – with bottom-up social perception – that is, a face-like stimulus. In four blocks, participants performed key presses either causing eye movements of a face or movements in a geometric field. For both, participants were made to believe to either be interacting with a partner, or to be performing the experiment by themselves. Time estimates were shorter for face-computer, face-confederate, and pattern-confederate conditions. Estimates for pattern-computer conditions were longest.

While these experiments [9,10] clearly demonstrate social hyperbinding based on evidence from both top-down and bottom-up manipulations, they lack the direct comparison between interaction (i.e., movements following actions) and passive observation (i.e., movements following the appearance of an arrow). To fill this gap, we conducted an experiment focused on the effects of a face stimulus versus a pattern stimulus during interaction and during observation. In accordance with social hyperbinding, we hypothesized that interaction with a face stimulus would cause time estimates to be shorter than during observation of a face stimulus and to be shorter than during the interaction with a pattern stimulus.

## 2. Method

In accordance with Vogel et al. [9,10], we used two different stimuli comprising a standardised face stimulus and a geometric stimulus consisting of the elements used to create the face stimulus (Fig 1). We presented stimuli in PsychoPy2 [28] on a 22-inch computer screen (resolution 1680 x 1050 pixels) against a standard grey background with a viewing distance of approximately 70 cm. Participants used a standard computer keyboard and mouse for their responses.

The stimulus material was selected according to Vogel et al. [9,10] and was highly schematic in nature (see Fig 1). To ensure the adequate identification of stimuli as faces and patterns, the block-wise instructions referred to the material specifically as “face” and “pattern”. Using different specific labels is important for the interpretation of stimuli, for example, [29] demonstrated that participants changed their behavior towards identical stimuli depending on whether they were referred to as either “gaze” or as “car”.

The paradigm resulted in a 2x2x2 factorial design. The factors manipulated were stimulus (face vs. pattern), agency (active vs. passive), and interval between initial event – either active key press or passive arrow observation – and its corresponding effect (400ms vs. 700ms). The experiment consisted of four blocks of 60 trials each. Stimulus and agency were presented by block in a crossover design. Intervals were randomized within blocks.

**Fig 2:**
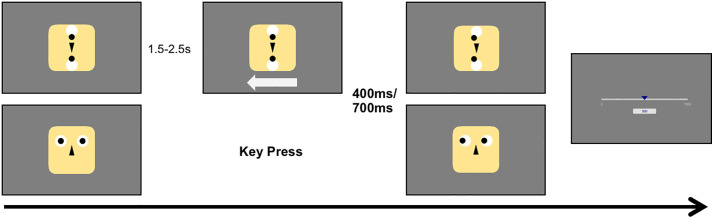
Exemplary trial event structure for passive/pattern trials (top row), and active/face trials (bottom row) as depicted in Vogel et al. (2021, 2022).

During active/pattern blocks, participants voluntarily pressed either a left or a right key on their computer keyboard at the time of their choosing (Fig 2). 400ms or 700ms after the key press the black dots in the pattern stimulus moved according to the indicated direction.

During passive/pattern block trials, participants were presented with the pattern stimulus (Fig 2). After between 2.5s to 3.5s a white arrow pointing either to the left or to the right appeared beneath the stimulus. Either 400ms or 700ms after the appearance of the arrow the two black dots in the pattern moved to the direction indicated by the arrow.

After both active and passive trials, a visual analogue scale appeared on screen ranging from 0 to 1000ms. With their computer mouse participants used the scale to provide an estimate of the interval between the cause (active, passive) and its effect (face, pattern).

For passive/face and active/face blocks the pattern stimulus was replaced by the face stimulus which moved its eyes represented by black dots in accordance with the initial events’ direction (arrow direction or key press direction).

Participants performed 60 trials per block, 30 with 400ms intervals and 30 with 700ms intervals, randomized across blocks. We counterbalanced blocks across participants. To guarantee full comparability with previous experiments [9,10] dots/eyes moved on every sixth trial to the opposite direction than indicated by the initial event. Due to the profound impact of highly schematic averted-gaze faces – such as the stimuli used in this study – on attention and interaction [30], these fail trials were excluded from data analysis.

All procedures performed were in accordance with the ethical standards of the institutional and/or national research committee and with the 1964 Helsinki Declaration and its later amendments or comparable ethical standards. The study was approved by the Ethics Committee of the Medical Faculty of the University of Cologne (No. 17–349). Written informed consent was obtained from all individual participants included in the study. All participants were naïve as to the purpose of the experiment. Participants received 10€ per hour as compensation for their participation. The recruitment period started on August 28^th^ 2023 and ended on September 28^th^ 2023.

## 3. Results

Based on the effect sizes reported by Vogel et al. (2021) [9], we initially conducted an a priori power analysis in G*Power [31] for paired-samples comparisons with ⍺ = 0.05, desired power = 0.85, Cohen’s dz = 0.68, yielding a minimum target sample size of 22 participants. To account for modifications to the original paradigm and the use of linear mixed-effects modelling, we deliberately exceeded this estimate and recruited 40 participants (16 identifying as male, 24 identifying as female; mean age 28.2, SD 6.85).

We planned to conduct a linear mixed effects model as recommended for repeated measures designs [32,33] on participants’ duration estimates. Data were analyzed using the R-based software jamovi and the GAMLj module [34–36]. We plotted figures in R-studio [37]. The duration estimates in milliseconds constituted the continuous dependent variable. Jamovi’s Linear Mixed Models module GAMLj uses R’s lme4 package to fit models [38]. We aligned the linear mixed model structure according to the experimental structure, with agency, stimulus, and interval as fixed effects, and participant ID as random effect, specified as random intercepts. To determine the model’s random structure we adopted the simplification approach by Barr et al. [39], starting with the maximal random structure and dropping random slopes until the model converged. We opted for a maximum random structure to emphasize the replicative nature of the experiment and to minimize type I error probability.

The final model structure was *Estimate ~ 1 + Agency + Stimulus + Interval + Agency:Stimulus + Agency:Interval + Stimulus:Interval + Agency:Stimulus:Interval+(1 + Agency + Stimulus + Interval + Agency:Stimulus + Agency:Interval + Stimulus:Interval | ID)*. The model was fitted using Restricted Maximum Likelihood (REML) and the Bound Optimization by Quadratic Approximation (bobyqa) optimizer. The final model successfully converged. Degrees of freedom for the fixed effects were computed using the Satterthwaite approximation. Significance thresholds were set at 0.05.

The model explained a substantial portion of the variance (R2(marginal) = 0.203; R2(conditional) = 0.626), with visual residual diagnostics indicating minor, non-severe deviations from normality and homoscedasticity.

We found significant differences between estimates for the factor interval (*700ms - 400ms*: F(1, 39)=123.845; M = −187.25ms, SE = 16.83ms, t = 11.13, p < 0.001). We detected a trend towards significance for the factor stimulus (*face – pattern*: F(1, 39) = 3.685; M = −16.35, SE = 8.52, t = −1.92, p = 0.062). The main effect of agency was not significant (*active – passive*: F(1, 39)=0.669; M = −10.79, SE = 13.19, t = −0.818, p = 0.418), confirming the null hypothesis for an emergence of TB in this sample.

The interaction between agency and stimulus showed a trend towards significance (active – passive * face – pattern: F(1, 39)=3.324; M = −27.31, SE = 14.98, t = −1.823, p = 0.076). The interactions with the factor interval, including the three-way interaction, were not significant, (agency * interval: F(1, 39)=0.704, M = 9.2, SE = 11.02, t = 0.839, p = 0.407; *stimulus × interval*: F(1, 39)=2.900, M = 12.35, SE = 7.25, t = 1.703, p = 0.097; agency * stimulus * interval: F(1, 7719)=0.363; M = −6.91, SE = 11.47, t = −0.602, p = 0.547)

**Fig 3:**
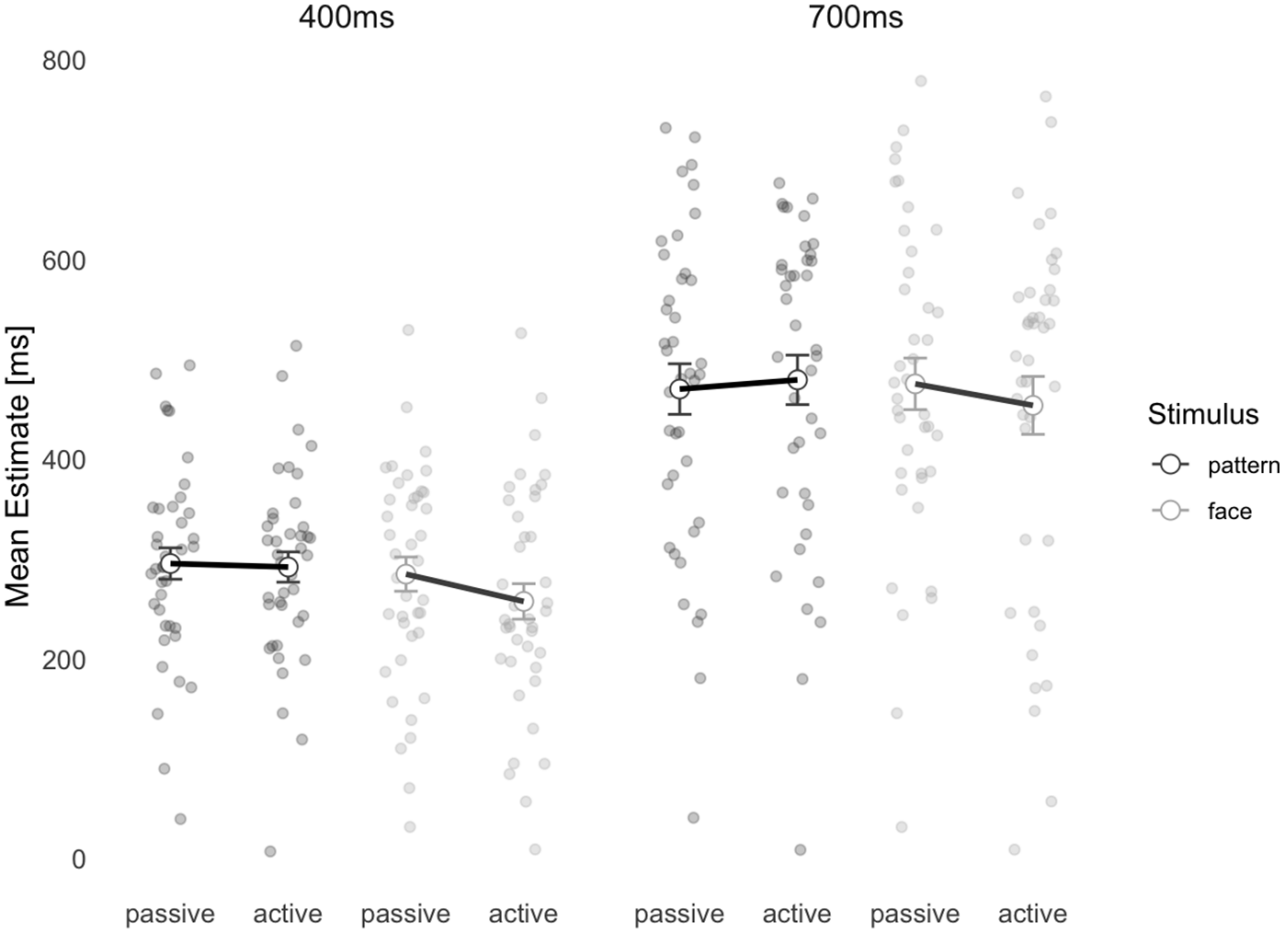
Mean time estimates for 400ms durations (left) and 700ms durations (right). Participant means for the pattern stimulus are depicted in the first two columns on each side in dark grey; participant means for the face stimulus are depicted in light grey in the two columns to their right. Data points on the left represent passive conditions during which participants observed durations between the appearance of an arrow and a subsequent movement in the presented stimulus. Data points on the right represent time estimates for active conditions during which participants actively caused the stimulus movement by pressing a button. Note the reduction in temporal estimates in the condition active/face for both durations (400ms, 700ms). Error bars depict standard error (SE).

Post-hoc comparisons for the Agency × Stimulus interaction collapsed across interval revealed no Bonferroni-corrected significant effects. The comparison between active pattern and active face was significant before correction (M = 30.01, SE = 12.90, t(39) = 2.326, p = 0.025), but not after Bonferroni correction (p_bonf_ = 0.152). All remaining contrasts were non-significant both before and after correction (*active/pattern vs. passive/face*: M = 5.56, SE = 14.57, t(39) = 0.382, p = 0.705, p_bonf_ = 1.000; *passive/face vs. active/face*: M = 24.44, SE = 15.32, t(39) = 1.595, p = 0.119, p_bonf_ = 0.712; *passive/pattern vs. active/face*: M = 27.14, SE = 16.75, t(39) = 1.620, p = 0.113, p_bonf_ = 0.680; *passive/pattern vs. active/pattern*: M = −2.87, SE = 15.01, t(39) = −0.191, p = 0.849, p_bonf_ = 1.000; *passive/pattern vs. passive/face*: M = 2.69, SE = 9.53, t(39) = 0.283, p = 0.779, p_bonf_ = 1.000) Fig 3.

Overall these result are similar to the two earlier studies [9,10] in magnitude, yet the analysis did not yield statistically significant results for the effects and interactions in question. In particular the data did not reveal social hyperbinding. Table 1 lists the relevant values from all three studies for comparison.

**Table 1:** Magnitude of Social Hyperbinding Across Studies and Experiments. Mean Binding Difference (ms) reflects the contrast between social and non-social conditions within each paradigm. In full operant–observant designs (Vogel et al., 2021, Exp. 1; Vogel et al., 2022, Exp. 1), this corresponds to the *Agency × Partner* interaction, where Partner denotes whether the action outcome was depicted by a face stimulus and believed to be produced by another person or was depicted as a pattern believed to be produced by a computer. In operant-only designs (Vogel et al., 2021, Exp. 2), the relevant contrast is the Stimulus effect (Face vs. Pattern), indexing bottom-up social cue processing as targeted by the present study. In belief-manipulation paradigms (Vogel et al., 2022, Exp. 2), the effect corresponds to *Agency × Story*, where Story reflects whether participants believed they were interacting with a human agent or a computer system while the action outcome was always depicted as a moving pattern stimulus. In the current study, the relevant contrast is *Agency × Stimulus* (Face vs. Pattern), capturing the interaction between action and social perceptual cues. Positive values indicate stronger temporal binding in social relative to non-social conditions. For Vogel et al. (2022), who investigated TB in autism spectrum disorder (ASD), values from typically developed participants and ASD values are simple-effect estimates derived from the reported mixed-model coefficients; inferential statistics correspond to the fixed effects reported in the original models. ANOVA results are reported as F-statistics; mixed-model results are reported as t-statistics.

Study	Experiment	Sample	Sample Size	Effect of Interest	Analysis Strategy	Mean Binding Difference (ms)	Test Statistic	P-values
*Vogel et al. (2021)*	Exp. 1	Typically Developed	24	Agency × Partner	Repeated measures ANOVA	≈ 60	F(1,23) = 11.02; ƞ2 = 0.324	.003
	Exp. 2 (operant only)	Typically Developed	32	Stimulus (Face vs. Pattern)	Repeated measures ANOVA	≈ 30	F(1,31) = 5.85	.022
*Vogel et al. (2022)*	Exp. 1	Full sample (Typically Developed + ASD)	48	Agency × Partner	Linear Mixed Model	33.24	t = −5.380	<.001
		Typically Developed	24	Agency × Partner		33.24	—	—
		ASD	24	Agency × Partner		6.62	—	—
				Agency × Partner × Group		—	t = 2.154	.031
	Exp. 2	Full sample (Typically Developed + ASD)	45	Agency × Story	Linear Mixed Model	40.70	t = −6.834	<.001
		Typically Developed	22	Agency × Story		40.70	—	—
		ASD	23	Agency × Story		15.30	—	—
				Agency × Story × Group		—	t = −2.133	.033
*Current Study*	—	Typically Developed	40	Agency × Stimulus	Linear Mixed Model	27.31	t = −1.823	.076

To evaluate the sensitivity of the present design, we conducted a post-hoc simulation-based power analysis using jamovi’s pamlj module [40] for the final linear mixed-effects model using the observed fixed and random effect structure and the final sample size (N = 40; 8000 observations total). The simulation indicated excellent power for detecting the main effect of interval (1-β = 1.00). However, the power for the main effects of agency and stimulus was low (1-β = 0.14) to moderate (1-β = .51), respectively. The power for the stimulus × interval interaction (1-β = 0.74) and the agency × stimulus interaction – the primary effect of theoretical interest regarding social hyperbinding – was moderate (1-β = 0.48), while the power for the agency × interval interaction was low (1-β = .15). These findings suggest that although the present design was sufficiently sensitive to detect large interval-related effects, it had a reduced sensitivity for smaller agency-related interaction effects. Consequently, the absence of statistically significant evidence for social hyperbinding in the present study should be interpreted cautiously, as smaller interaction effects may have remained undetected.

## 4. Discussion

We conducted a temporal binding paradigm in order to complement earlier findings on social hyperbinding. Previous studies had indicated a pronounced TB effect for social interaction [5,6 7,8,11,12], with some suggesting a similar effect for face stimuli [9,10,17,18]. In summary, we failed to convincingly reject the null hypothesis of binding for the active manipulation of faces. While the data indicated a visible trend towards an occurrence of shorter time estimates primarily for interactions with a face stimulus – suggestive of hyperbinding – only the differentiation of durations and none of the other effects and interactions surpassed the preset significance threshold.

This contradicts earlier hypotheses on a ubiquitous social hyperbinding independent from whether social information is presented as a belief in the presence of another person or is presented as a social stimulus [9,10]. Contrary to our initial hypotheses and prior findings reported by Vogel et al. [9,10], our study cannot provide statistically significant evidence for either an effect of agency or of a face stimulus on time perception. The only statistically significant finding in our data was the main effect of interval, reflecting participants’ general sensitivity to differences in temporal duration.

These null findings call into question whether the previously reported effects of social hyperbinding, that is enhanced TB during social interaction, can be robustly replicated in the absence of additional factors such as belief in human agency or social engagement. Unlike in Vogel et al. [9,10], our experiment did not include a social cover story or explicit interpersonal framing. The lack of significant effects in the present study may suggest that social hyperbinding, to the extent that it occurs, may require more than just the perceptual presence of a face-like stimulus.

It is important to note that the absence of any reliable TB effect in our data – regardless of condition – is itself potentially meaningful. It suggests that the underlying conditions for TB were not met in this paradigm. If no temporal binding is elicited in the baseline condition, then modulations of that binding by stimulus type or agency become theoretically and statistically less likely. This absence may reflect boundary conditions for the TB effect, particularly in paradigms using temporal magnitude estimation without anchoring or feedback [41,42].

The cognitive demands of our task may have contributed to this outcome. Participants judged durations across repeated trials with varying action-outcome delays, while also being presented with face-like and abstract stimuli. These dual demands on attention and cognition could plausibly disrupt the implicit temporal compression in TB. TB is typically strongest under conditions of high temporal predictability [25–27] and low cognitive load [43,44]; when task effort increases TB may be weakened or masked. Thus, our null finding may reflect a genuine absence of binding under cognitively demanding conditions, rather than an artifact of low power or insufficient manipulation.

It remains possible that social stimulus material may not strengthen TB, but may in fact weaken or disrupt it under certain conditions. The presence of socially salient stimuli, such as faces, could introduce competing attentional or cognitive demands that interfere with the processes underlying TB. Notably, Ulloa et al. [18], while reporting shortened duration estimates in response to gaze shifts of naturalistic face stimuli, did not include a passive control condition in their design. Without this comparison, it is difficult to determine whether the observed effect reflects increased TB in the social condition or a general reduction in temporal estimates due to social salience. Our data, which included both active and passive conditions, did not show such a specific difference between active and passive conditions. This would further suggest that social stimuli alone are insufficient to produce reliable TB effects and could in some cases even attenuate them. However, this serves as an alternative hypothesis, as a possible limitation or flaw in our paradigm might better explain our results.

Several limitations of our study may have contributed to the null effects. The task we used – a temporal magnitude estimation task – was not optimized for precise time estimation and is subject to individual anchoring biases [10,41,42]. In addition, we did not include a manipulation check to confirm that participants perceived stimuli as intended (meaning as faces vs. patterns). While schematic faces may evoke attentional processes comparable to those triggered by realistic faces [45], we cannot guarantee that such attentional effects transfer to our paradigm. This potential deficit in ecological validity may have negatively influenced effect sizes by reducing emotional intensity or activation of mentalizing or mirroring processes [46], subsequently resulting in a perceptual difference between stimuli too subtle to elicit a measurable effect in time estimation. These factors limit the interpretability of any trends and highlight the need for further rigorous paradigms in future research.

Overall, our data do not replicate the key interaction effects between agency and social stimulus that have been interpreted as evidence for social hyperbinding. While prior studies suggest that such effects may arise under certain conditions – particularly when involving social beliefs [9,10] – our results provide no evidence for their occurrence in the current paradigm. Future studies should investigate the boundary conditions under which TB and social hyperbinding may or may not emerge, ideally using designs that further isolate and compare the contributions of perceptual, attentional, and belief-driven factors.

The results of this study emphasize the question about the direct relationship between social perception and social belief. The examination of how interacting with an object of non-human appearance in the belief in a human interactant controlling this object relates to interacting with an object of human appearance knowing that it is not controlled by another person might provide important insight into the relationship between bottom-up and top-down social cognition. Speculatively, as previous work [10], replicated social hyperbinding for social belief without face stimuli (see Table 1), yet our current study did not replicate social hyperbinding for face stimuli without social belief, the introduction of a cover story might elicit increased TB where a social stimulus might not.

In conclusion, although our study was motivated by previous findings suggesting an interplay between agency, social stimuli, and time perception, the present data do not support these effects. We found no significant evidence for social hyperbinding or agency-driven TB for social perception alone, and thus our results contribute to an increasingly nuanced picture of when and how social context influences human time perception and TB. Replication efforts with higher statistical power, manipulation checks, and a broader range of social stimuli are needed to more definitively assess the reliability and generalizability of these effects.
