# Supplementary material for: Temporal binding and social perception – A replication study
Source: PLoS One. 2026 Jul 14;21(7):e0352510. doi: 10.1371/journal.pone.0352510 (PMC13367721; doi:10.1371/journal.pone.0352510)
Supplement: Attachment — Submitted filename: 26-03-18_rebuttal_V1.2.docx [file pone.0352510.s002.docx]

**Reviewer Comments to the Author — Point by Point Reply:**

**Reviewer #1: This study investigated whether temporal binding is enhanced by social perception, specifically by interacting with a face stimulus. Participants performed a time estimation task in active and passive conditions using face and non-social pattern stimuli. Although a trend toward shorter time estimates was observed when participants actively interacted with a face, the results did not provide reliable evidence for temporal binding or social hyperbinding driven by face perception alone.**

Overall, the study is well conducted and clearly fits the aims and scope of the journal (in this case, publishing null results). I have only a few minor comments that I believe could help to further improve the clarity and interpretation of the manuscript.

*We appreciate the reviewer’s positive feedback.*
—————————————————————

**One important limitation concerns the low ecological validity of the stimuli. Although it is well established that highly schematic faces can elicit social attentional processes comparable to those triggered by real faces - please refers to a recent evidence; (Dalmaso et al., 2025) - there remains some uncertainty about how participants actually perceived the different stimuli in the present study. In this respect, the absence of a manipulation check represents a limitation, as such a measure could have helped to assess whether the face stimuli were indeed perceived as socially meaningful and distinct from the pattern stimuli. I would therefore encourage the authors to expand the Discussion by explicitly addressing the issue of ecological validity and stimulus perception, and by considering how this aspect may have contributed to the null results.**

*We thank the reviewer for the close reading of our manuscript and agree that in its current form the limitation brought on by the schematic nature of the stimulus material falls short. We appreciate the addition of the work by Dalmaso et al. (2025), which we had not been aware of to date and now feature in the manuscript. We have amended the discussion section to more thoroughly develop the limitation. The paragraph on page 9 now reads:*

“In addition, we did not include a manipulation check to confirm that participants perceived stimuli as intended (i.e., as faces vs. patterns). While schematic faces may evoke attentional processes comparable to those triggered by realistic faces (Dalmaso et al., 2025), we cannot guarantee that such attentional effects transfer to our paradigm. This potential deficit in ecological validity may have negatively influenced effect sizes by reducing emotional intensity or activation of mentalizing or mirroring processes (Vogeley, 2017), subsequently resulting in a perceptual difference between stimuli too subtle to elicit a measurable effect in time estimation. These factors limit the interpretability of any trends and highlight the need for further rigorous paradigms in future research.”

——————————————————

**I would appreciate some clarification regarding the power analysis. The sample size seems to have been determined using GPower based on a paired-samples t-test effect size, while the data were analysed using linear mixed-effects models. I would therefore welcome clarification on how this power calculation maps onto the mixed-model framework and the specific effects of interest (e.g., interactions), and whether it provides an adequate estimate of statistical power for the reported analyses.**

*We thank the reviewer for this important point and appreciate the opportunity to clarify our power analysis.*

*The a-priori power analysis was conducted in G*Power based on a paired-samples t-test because the only available prior study closely matching our experimental paradigm reported its results using a repeated-measures ANOVA framework (Vogel et al., 2021). The reported effect size was converted to Cohen’s d and used to estimate the required sample size. The selected effect most closely corresponded to the within-subject contrast central to our primary hypothesis. Although our confirmatory analyses were conducted using a linear mixed-effects model, the powered contrast corresponds directly to a fixed effect in the mixed-model framework. We have added the following clarification to the manuscript on page 6:*

"We acknowledge that power analyses based on t-tests do not fully account for the additional variance components estimated in LMMs (e.g., random intercepts and slopes). However, given the fully within-subject design and balanced structure, and because the effect size was derived from a comparable paradigm (Vogel et al., 2021), we consider this approach a reasonable and conservative approximation at the planning stage. Importantly, the final sample size exceeded the minimum estimated by the a-priori power analysis.”

—————————————————————————-

**Lastly, I was unclear about the reasons for not sharing the data publicly. Given that the study appears to involve standard behavioural measures (time estimates, experimental conditions, anonymous participant IDs), it is not obvious which privacy concerns would prevent the release of a fully de-identified dataset. I would encourage the authors to clarify this point and, if possible, to consider making the data publicly available in line with open science and replication practices (this is particularly relevant for a journal like Plos One).**

*We wholeheartedly agree with the reviewer. After consultation with both the approving ethics committee, our data security officer, and the journal editor we are now able to publish the data at our discretion.*

*The data are available at: osf.io/vpyd9*

**Reviewer #2:** **In their study, the authors attempt to replicate findings from previous experiments, where they found a temporal hyperbinding of actions and social effects. In particular, participants reported shorter time intervals between actions and effects they believed to represent another human’s behaviour and were represented on a face like stimulus as compared to a geometrical pattern that was not related to another human’s behaviour. In the current study they contrasted the two stimuli without a social cover story and found no significant temporal binding at all.
I commend the authors for transparently reporting their null results! Exploring the specific mechanisms and boundary conditions of temporal binding is, I believe, a very current issue which is why these results are very much worth publishing.
However, the lack of raw data and without any (reported) exploratory tests, it is difficult to understand the reason, why the previously found temporal binding effect was absent here.**

*We thank the reviewer for their appreciation of our results and fully agree with the stated relevance of boundary conditions to temporal binding (TB) and psychological effects in general. In the following, we will address the important issues the reviewer raises.*

—————————————

**Major Point 1:
If I understand correctly, the face condition in the current study is identical to the one used in Experiment 1 of Vogel et al. (2021). The only change seems to have been made to the pattern condition, where the movement was adapted to more closely match the movement of the face stimulus. The conditions in Experiment 1 of your study Vogel et al. (2022) are fully identical with the only exception that both original experiments include a cover story, which relates the eye movements to a real person. Despite the face conditions being “on the face of it” identical in all sets, the original studies reported clear temporal binding (TB), whereas the present data do not.**

*We appreciate the reviewer’s appreciation of our work. Contrary to the reviewer’s understanding, we must point out that the experiments referred to in this comment were identical, but differ from the experiment described in this study. Both the first experiments in Vogel et al., 2021 and 2022 entailed a combination of a face stimulus and a cover story, thereby confounding these two aspects of sociality. In contrast, the current study attempts to clarify the true nature of the influence of face/gaze perception on TB. This confusion underscores the reviewer’s following points which we addressed in a new table 1 explained and depicted below.* ***Table 1*** *includes a column (named “Effect of Interest”) dedicated at stimulus/cover story combinations in these studies.*

**—————————————**

**Several interpretations of this come to mind which probably each warrant a separate response. Nevertheless, they all pertain to the relationship between this data and the one from your original study, and may also, especially if I misunderstood or overlooked something, be answered together:

Major 1A: The sample sizes differ. The earlier experiments had a smaller sample (of neurotypical participants), than the current experiment. Although temporal binding often shows a robust mean effect, interindividual differences are notoriously high. It would therefore be informative to examine whether the effect survives a pooled analysis of the three samples. Since this paper is to be published as a stand-alone, I think it would be helpful to include the results you are trying to replicate in one of the graphs. Right now, I had to circle back to your original studies to really grasp the reasoning and the key differences. As figures are quite different between the three manuscripts, a unified depiction would help me (and presumably others) a lot.**

*We thank the reviewer for this thoughtful and constructive suggestions. We agree that a clearer, unified depiction of the previously reported effects improves transparency and makes the manuscript more self-contained.*

*To address this point, we have now added* ***Table 1****, which summarizes all relevant experiments from Vogel et al. (2021, 2022) alongside the present study. The table reports the specific effect of interest in each paradigm (i.e., Agency × Partner, Agency × Story, Agency × Stimulus), the corresponding mean temporal binding difference, and the original inferential statistics. For the 2022 study, we additionally report group-specific (TD and ASD) effect magnitudes derived from the published mixed-model coefficients, as well as the formal test of group moderation.*

*This unified presentation will allow readers to directly compare effects across paradigms and samples without having to consult the earlier manuscripts. It includes the mean social hyperbinding effect. The magnitude observed in the present study (27.31ms) falls within the previously reported range, albeit not reaching statistical significance.*

*Regarding the suggestion of a pooled analysis, the prior studies differ in design, rendering a direct pooled model across all datasets statistically inappropriate for a replication study. We therefore opted for a structured comparative summary of effect magnitudes and statistical tests across experiments, which we believe more accurately reflects the replication logic.*

*We hope that this addition improves clarity and addresses the reviewer’s concern about robustness and variability. Please see the added* ***Table 1*** *in the attached file.*

————————————————————-

**Major 1B: The analysis strategy also differed between studies. In your 2021 study, ANOVAs were conducted, whereas the 2022 and the present study uses a mixed model with Subject ID as a random factor. I wonder what motivated you to switch from ANOVAs to mixed models. Honestly, I do not assume that an ANOVA would have given you any different results here. Nevertheless, a direct comparison of both analyses on both datasets would be interesting. As far as I understand, you did do a t-test on the specific contrast already but never reported it.**

*We thank the reviewer for this thoughtful comment. The shift from repeated-measures ANOVA (2021 study) to linear mixed-effects models (2022 and present study) reflects an evolution in our analytic approach rather than a conceptual change. Mixed-effects models allow us to analyze trial-level data without aggregation, appropriately model subject-level variability, and handle potential imbalance or missing data more flexibly. For these reasons, they are now generally recommended for within-subject designs of this kind. However, and as mentioned in your introduction of Major 1, we believe point Major 1B to be complementary to Major 1A. We hence list Test statistics results in Table 1.*

*————————————————-*

**Major 1C: Modifying the movement of the pattern stimulus may have influenced the overall interpretation of the stimuli, including the perception of the face stimulus. In my opinion, the pattern stimulus has more than just a little resemblance to a sideways face. May the critical factor in the earlier experiment have been not the “socialness” of the face stimulus per se, but rather the perceived socialness of the effect (moving eyes vs shifting overall position). If I read your papers correctly, this is not the case in the 2022 study, but still, the face is associated with a real person here and the pattern is not. I wonder what the authors would predict if the reinstated their cover story for the pattern but not the face. Or if they had the whole face move (like the pattern in the 2021 study) while the pattern only moved the black dots.
I understand completely that the main problem was a lack of temporal binding altogether. Unless it was pure chance, I still feel like there is much potential here to disentangle the influencing factors and possibly find the relevant conditions that diminish temporal binding.**

*We thank the reviewer for this highly interesting suggestion concerning further paradigm changes and social binding effects! A comparison between the purely perceptual versus the belief socialness — in other words comparing pattern/story to face/no-story stimuli — would shed further light on the relationship between both aspects of social hyperbinding. Two competing potential results come to mind: a) the null hypothesis with a similar result as seen in the current study; b) increased binding for the pattern/story combination. We strongly favor the latter — particularly in a within subjects design — as in our opinion the previous studies suggest a superiority of or preference for social belief in social temporal binding causation. However, this would require an additional experiment and is beyond the scope of this replication study.*

*We have added this intriguing idea to the discussion section’s paragraph about future investigations on page 9. We also believe that Table 1 increases readability of this intricate research design. The rewritten paragraph now reads:*

“The results of this study emphasize the question about the direct relationship between social perception and social belief. The examination of how interacting with an object of non-human appearance in the belief in a human interactant controlling this object relates to interacting with an object of human appearance knowing that it is not controlled by another person might provide important insight into the relationship between bottom-up and top-down social cognition. Speculatively, as previous work (Vogel et al, 2022), replicated social hyperbinding for social belief without face stimuli (see Table 1), yet our current study did not replicate social hyperbinding for face stimuli without social belief, the introduction of a cover story might elicit increased TB where a social stimulus might not.”

*———————————————-*

**Major Point 2:
I encourage the authors to report the mean effect for agency, even if (or especially because) it is not statistically significant. It would also be helpful to know whether there is significant temporal binding in the social condition alone. Once again, I am convinced you must have done this test in the “other t-tests that were not significant”, but please share them with the readership.
More generally, I recommend reporting the mean effects and effect sizes for all primary comparisons, along with the results of all follow-up t-tests - simply all tests that you performed. To keep the manuscript slim, you could consider including a comprehensive table of all tests and results in an online repository.**

*We thank the reviewer for this helpful suggestion to improve readability and replicability. We have extended our results section by the requested information. We kept the information briefly structured and do not believe it surpasses a readable manuscript length. We added some minor information to the already mentioned results and added the following paragraph on page 7:*

*“*The interaction between agency and stimulus showed a trend towards significance (*active - passive * face - pattern*: F(1, 39)=3.324; M=-27.31, SE = 14.98, t=-1.823, p=0.076). The interactions with the factor interval, including the three-way interaction, were not significant, (*agency * interval*: F(1, 39)=0.704, M=9.2, SE = 11.02, t=0.839, p = 0.407; *stimulus × interval*: F(1, 39)=2.900, M=12.35, SE=7.25, t=1.703, p=0.097; *agency * stimulus * interval*: F(1, 7719)=0.363; M=-6.91, SE=11.47, t=-0.602, p=0.547)
Post-hoc comparisons for the Agency × Stimulus interaction collapsed across interval revealed no Bonferroni-corrected significant effects. The comparison between active pattern and active face was significant before correction (M = 30.01, SE = 12.90, t(39) = 2.326, p = 0.025), but not after Bonferroni correction (p_bonf_ = 0.152). All remaining contrasts were non-significant both before and after correction (*active/pattern vs. passive/face*: M = 5.56, SE = 14.57, t(39) = 0.382, p = 0.705, p_bonf_ = 1.000; *passive/face vs. active/face*: M = 24.44, SE = 15.32, t(39) = 1.595, p = 0.119, p p_bonf_ = 0.712; *passive/pattern vs. active/face*: M = 27.14, SE = 16.75, t(39) = 1.620, p = .113, p p_bonf_ = 0.680; *passive/pattern vs. active/pattern*: M = −2.87, SE = 15.01, t(39) = −0.191, p = 0.849, p p_bonf_ = 1.000; *passive/pattern vs. passive/face*: M = 2.69, SE = 9.53, t(39) = 0.283, p = 0.779, p p_bonf_ = 1.000).*”*

———————————————————————

**Major Point 3:
Speaking of online repositories, I am not persuaded by the authors’ justification for not sharing their data openly. It sounds very much like a standard phrase as there are no patients included in this study. I strongly urge the authors to share their (anonymized) raw data and analysis scripts on a platform such as the OSF. Open sharing reduces barriers to replication, reanalysis, and meta-analyses. Providing data on demand has multiple disadvantages, as authors can become uncontactable, and data may be lost on local machines. While open platforms are not perfect, they provide an additional layer of security and accessibility for valuable datasets. Please seriously consider this and if it’s not an option for you, please provide a more specific explanation why not for this data set.
In any case, I would request the authors to share the anonymized raw data and analysis script of this experiment as well as the previous studies (at least Experiment 1 from both) with me and all other reviewers for the next round of reviews.**

*We wholeheartedly agree with the reviewer. After consultation with both the approving ethics committee, our data security officer, and the journal editor we are now able to publish the data at our discretion.*

*The data for this study are available at: osf.io/vpyd9*

*The data for Vogel et al., 2021 are available at: osf.io/ncrkv*

*The data for Vogel et al., 2022 are available at: osf.io/ajbe6*

———————————————————-

**Minor Points
• The statement that temporal binding is strongest under “high temporal predictability and low cognitive load” (ll. 244-245) and may be weakened or masked when task complexity increases, reads as somewhat contrived. While the claim may be correct, it is not self-evident. Please provide supporting citations.**

*Thank you for bringing this to our attention. We now present original work supporting this proposal , namely Cravo et al., 2011; Ruess et al., 2017; Klaffehn et al., 2021; Howard et al., 2016; Qu et al., 2021.*

————————

**• Additionally, the claim that social information “may draw attention away from temporal processing, leading to compressed duration estimates” (ll.265-266) seems quite at odds with the earlier argument that temporal binding is strongest under low cognitive load.**

*We agree with the reviewer’s statement and wish to point out that while the manuscript line mentioned in the previous comment serves as an interpretation of the null finding, this second line is intended to provide an explanation for the statistical trend for the interaction. We interpret the reviewer’s comment as indicative that this second line of argument concerning a statistical trend may cause confusion with readers. Hence, we have removed the entire paragraph from the resubmitted manuscript.*

*—————————————*

***• The statement that “social stimulus material may not strengthen temporal binding, but may in fact weaken or disrupt it under certain conditions” (ll. 249-250) does not convince me as currently argued. In your experiment, the difference between passive and active trials was smaller, descriptively reversed even, in non-social trials. Additionally, the social connotation in your original experiments was much stronger. Would it not make more sense to consider a possible limitation or flaw in the current experimental design rather than concluding that social stimuli weaken temporal binding?***

*We agree with the reviewer’s argument and wish to emphasize that our corresponding argument is meant as an alternative explanation to our results. We have added this to the manuscript to better make clear the hypothetical and arguably unlikely nature of this circumstance on page 8:*

“However, this serves as an alternative hypothesis; a possible limitation or flaw in our paradigm might better explain our results.”

————————

**• The argument beginning with “Given these null results, we urge caution…” at l. 258 - 261 is a bit repetitive.**

*The argument was deleted in the revision process.*

————————————

**• The manuscript contains frequent use of “e.g.” and “i.e.”, particularly in the introduction. At least according to APA standard, these should only be used in parentheses. Personally, I find this rule sensible, as these abbreviations disrupt the reading flow so to speak. Of course this is just for your consideration.**

*Thank you for pointing out this often overlooked impediment to readability. With the exception of two instances we have changed all abbreviations.*

*————————————*

**Overall, it is an interesting manuscript and especially the reporting of null results aids the current discourse. Still, the discussion is quite ambiguous, as the authors seemingly (and understandably) didn’t really know what to make of the surprising lack of temporal binding. I would be interested in a few more substantiated ideas or exploratory analyses on this, for example in relation to the questions posed in Major Point 1.
I am very much looking forward to reading the authors’ thoughts on this.

Best wishes!
Annika Klaffehn**

*We thank the reviewer for the positive feedback. We have answered all above points and believe the manuscript has greatly improved from the constructive feedback. We are looking forward to any further comments and suggestions.*
