# Supplementary material for: Temporal binding and social perception – A replication study
Source: PLoS One. 2026 Jul 14;21(7):e0352510. doi: 10.1371/journal.pone.0352510 (PMC13367721; doi:10.1371/journal.pone.0352510)
Supplement: Attachment — Submitted filename: 26-05-15_V5.2-rebuttal.docx [file pone.0352510.s003.docx]

**Reviewer #2:**

**Dear Authors**

**Thank you for uploading the raw data. While I admit I was a bit disappointed that you did not share my enthusiasm for the exploratory cross-experiment analyses, which I would have found worthwhile despite the differences in cover stories, I do understand the reasoning. In any case, making the full dataset available largely addresses this concern, as interested readers can carry out these analyses themselves. I also appreciate your explanation regarding the discussion and the somewhat loosely connected set of hypotheses. Although the revisions in this section appear modest, in my opinion the text reads more clearly now. In particular, the section summarizing the study’s limitations is very helpful. Thank you as well for including a table with the summary statistics, which, as you put it, makes the article more self-contained. Overall, I believe the article is almost ready for publication. While reading, I only noticed a few minor errors and some slightly awkward phrasings that seem to have been overlooked during internal review. Additionally, I would ask the authors to review their power analysis once more, as I was not totally convinced by the changes they made.**

*We thank the reviewer for the positive feedback. We too believe the changes recommended in the last round of reviews have greatly improved the manuscript, particularly concerning clarity. We apologize for any errors that persisted after the first review and have now taken great care of correcting them.*

**P. 2 “However, while watching another person perform the task [6] or when performing the task with another person [5, 8, 16], TB is shorter than while performing the task alone. “**

**“TB is shorter” seems very weird to me and left me wondering whether you actually wanted to say that ratings became shorter. I guess the TB effect became smaller or decreased would be clearer.**

*We agree with the reviewer that our choice of phrasing was confusing. We have changed the sentence to:*
“However, while watching another person perform the task [6] or when performing the task with another person [5, 8, 16], TB is smaller than while performing the task alone.”

**P.3 “For one, TB has been thought to depend on the Sense of Agency –“**

**I stumbled a bit over this sentence. I’m not a native speaker so feel free to completely ignore me if I’m wrong, but is “thought” correct here? I would have expected something like “considered”, “believed” or “theorized”.**

*We have changed the sentence to:*
“For one, TB has been theorized to depend on the Sense of Agency – […]”

**P. 4 “For half of the trials participants were made to believe they were interacting with another person, while during the other half, they were told to be performing the experiment by themselves.”**

**“…they were told to be performing the experiment by themselves.” does not seem correct. “…they were told they would be performing the experiment by themselves.”?**

*We thank the reviewer for pointing this out. We agree that the original phrasing was unclear and have revised the sentence to:*

“For half of the trials participants were made to believe they were interacting with another person, while during the other half, they were told they would perform the experiment by themselves.”

**p. 5 “resulting in a minimum sample size per group at n = 22.”**

**What are the groups you speak of here? Why did you not calculate an effect size for within subjects analyses, if that was your plan all along? It reads as if it was simply an error made at the time. If that is the case, it is understandable but to avoid confusion you could also simply say that you based your sample size on the effect size from Vogel et al. 2019 (and report this original effect size). The calculated required sample was 22 participants, then add your explanation that you deliberately overshot this estimate in order to account for changes. Crucially, you could skip the lengthy and slightly awkward explanation of why ANOVA based power analyses are more or less appropriate and just instead run a post hoc simulation based power estimation for the LMM with your final sample size and report how much power you ended up with.**

*We thank the reviewer for this helpful suggestion. We agree that the original wording regarding “groups” was imprecise in the context of a fully within-subject design and that the previous justification of the a-priori power analysis was unnecessarily extensive.*

*In the revised manuscript, we therefore simplified the description of the initial sample size estimation. As originally intended, we now state that the recruitment target was based on the effect size reported by Vogel et al. (2021) using a paired-samples framework appropriate for the original planning stage, and that we deliberately exceeded this estimate to account for modifications to the paradigm and analysis strategy. The revised section reads:*

*“*Based on the effect sizes reported by Vogel et al. (2021) [9], we initially conducted an *a priori* power analysis in G*Power [31] for paired-samples comparisons with ⍺ = 0.05, desired power = 0.85, Cohen’s dz = 0.68, yielding a minimum target sample size of 22 participants. To account for modifications to the original paradigm and the use of linear mixed-effects modelling, we deliberately exceeded this estimate and recruited 40 participants (16 identifying as male, 24 identifying as female; mean age 28.2, SD 6.85).*”*

*Importantly, following the reviewer’s recommendation, we additionally conducted a post hoc simulation-based power analysis for the final linear mixed-effects model using the observed fixed and random effect structure and the final sample size. We placed this analysis in the Results section and now report the achieved power for the relevant model effects. Because the Agency × Stimulus interaction constituted the primary effect of theoretical interest with regard to social hyperbinding, we specifically highlight that the achieved power for this interaction was moderate (1-β = .48). The section added at the end of the results section reads:*

*“*To evaluate the sensitivity of the present design, we conducted a post-hoc simulation-based power analysis using jamovi’s pamlj module [40] for the final linear mixed-effects model using the observed fixed and random effect structure and the final sample size (N = 40; 8000 observations total). The simulation indicated excellent power for detecting the main effect of interval (1-β = 1.00). However, the power for the main effects of agency and stimulus was low (1-β = 0.14) to moderate (1-β = .51), respectively. The power for the stimulus × interval interaction (1-β = 0.74) and the agency × stimulus interaction – the primary effect of theoretical interest regarding social hyperbinding – was moderate (1-β = 0.48), while the power for the agency × interval interaction was low (1-β = .15). These findings suggest that although the present design was sufficiently sensitive to detect large interval-related effects, it had a reduced sensitivity for smaller agency-related interaction effects. Consequently, the absence of statistically significant evidence for social hyperbinding in the present study should be interpreted cautiously, as smaller interaction effects may have remained undetected.*”*

**p. 7 “All remaining contrasts were non-significant both before and after correction (active/pattern vs. passive/face: M = 5.56, SE = 14.57, t(39) = 0.382, p = 0.705, pbonf = 1.000; passive/face vs. active/face: M = 24.44, SE =15.32, t(39) = 1.595, p = 0.119, p pbonf = 0.712; passive/pattern vs. active/face: M = 27.14, SE = 16.75, t(39) = 1.620, p = .113, p pbonf = 0.680; passive/pattern vs. active/pattern: M = −2.87, SE = 15.01, t(39) = −0.191, p = 0.849, p pbonf = 1.000; passive/pattern vs. passive/face: M = 2.69, SE = 9.53, t(39) = 0.283, p = 0.779, p pbonf = 1.000).”**

**I think there are a couple of lonely ps before some pbonfs in this paragraph.**

*The lonely ps have been removed.*

**p.8 “as a possible limitation of flaw in our paradigm”**

**it should be or, not of, right?**

The typeo has been corrected. Thank you for pointing this out.
