# Supplementary material for: Temporal binding and social perception – A replication study
Source: PLoS One. 2026 Jul 14;21(7):e0352510. doi: 10.1371/journal.pone.0352510 (PMC13367721; doi:10.1371/journal.pone.0352510)
Supplement: Peer review history

# Peer review: Decision Letter 0

**DOI:** 10.1371/journal.pone.0352510.r001

**Authors:** Elisa Scerrati

16 Feb 2026

PONE-D-25-67312

Temporal Binding and Social Perception – a Replication Study

PLOS One

Dear Dr. Vogel,

Thank you for submitting your manuscript to PLOS ONE. After careful consideration, we feel that it has merit but does not fully meet PLOS ONE’s publication criteria as it currently stands. Therefore, we invite you to submit a revised version of the manuscript that addresses the points raised during the review process.

Your paper has now been reviewed by two experts in the field, and I am grateful for their thorough and constructive feedback. Both reviewers recognize the value of transparently reporting null results on temporal binding and social perception. However, they raise important concerns that must be addressed in a revision. The primary issue concerns data sharing, as they both find your current justification for withholding data as insufficient and strongly urge you to share anonymized raw data and analysis scripts publicly, so do I. Additionally, both reviewers have provided detailed comments on methodological and statistical aspects that require systematic attention. These include: clarification of the power analysis, stimuli ecological validity considerations, complete reporting of all performed analyses, and deeper investigation of the relationship between current and previous findings. Please address each point systematically in your response letter.

Please submit your revised manuscript by Apr 02 2026 11:59PM. If you will need more time than this to complete your revisions, please reply to this message or contact the journal office at plosone@plos.org. When you're ready to submit your revision, log on to https://www.editorialmanager.com/pone/ and select the 'Submissions Needing Revision' folder to locate your manuscript file.

Please include the following items when submitting your revised manuscript:

- A letter that responds to each point raised by the academic editor and reviewer(s). You should upload this letter as a separate file labeled 'Response to Reviewers'.
- A marked-up copy of your manuscript that highlights changes made to the original version. You should upload this as a separate file labeled 'Revised Manuscript with Track Changes'.
- An unmarked version of your revised paper without tracked changes. You should upload this as a separate file labeled 'Manuscript'.

If you would like to make changes to your financial disclosure, please include your updated statement in your cover letter. Guidelines for resubmitting your figure files are available below the reviewer comments at the end of this letter.

If applicable, we recommend that you deposit your laboratory protocols in protocols.io to enhance the reproducibility of your results. Protocols.io assigns your protocol its own identifier (DOI) so that it can be cited independently in the future. For instructions see: https://journals.plos.org/plosone/s/submission-guidelines#loc-laboratory-protocols. Additionally, PLOS ONE offers an option for publishing peer-reviewed Lab Protocol articles, which describe protocols hosted on protocols.io. Read more information on sharing protocols at https://plos.org/protocols?utm_medium=editorial-email&utm_source=authorletters&utm_campaign=protocols.

We look forward to receiving your revised manuscript.

Kind regards,

Elisa Scerrati

Academic Editor

PLOS One

Journal requirements:

When submitting your revision, we need you to address these additional requirements.

1. Please ensure that your manuscript meets PLOS ONE's style requirements, including those for file naming. The PLOS ONE style templates can be found at

https://journals.plos.org/plosone/s/file?id=wjVg/PLOSOne_formatting_sample_main_body.pdf and

https://journals.plos.org/plosone/s/file?id=ba62/PLOSOne_formatting_sample_title_authors_affiliations.pdf

2. Please include a separate caption for each figure in your manuscript.

3. In the online submission form you indicate that your data is not available for proprietary reasons and have provided a contact point for accessing this data. Please note that your current contact point is a co-author on this manuscript. According to our Data Policy, the contact point must not be an author on the manuscript and must be an institutional contact, ideally not an individual. Please revise your data statement to a non-author institutional point of contact, such as a data access or ethics committee, and send this to us via return email. Please also include contact information for the third party organization, and please include the full citation of where the data can be found.

If the reviewer comments include a recommendation to cite specific previously published works, please review and evaluate these publications to determine whether they are relevant and should be cited. There is no requirement to cite these works unless the editor has indicated otherwise.

[Note: HTML markup is below. Please do not edit.]

Reviewers' comments:

Reviewer's Responses to Questions

**Comments to the Author**

1. Is the manuscript technically sound, and do the data support the conclusions?

The manuscript must describe a technically sound piece of scientific research with data that supports the conclusions. Experiments must have been conducted rigorously, with appropriate controls, replication, and sample sizes. The conclusions must be drawn appropriately based on the data presented.

Reviewer #1: Yes

Reviewer #2: Partly

**********

2. Has the statistical analysis been performed appropriately and rigorously?

Reviewer #1: Yes

Reviewer #2: Yes

**********

3. Have the authors made all data underlying the findings in their manuscript fully available?

The PLOS Data policy requires authors to make all data underlying the findings described in their manuscript fully available without restriction, with rare exception (please refer to the Data Availability Statement in the manuscript PDF file). The data should be provided as part of the manuscript or its supporting information, or deposited to a public repository. For example, in addition to summary statistics, the data points behind means, medians and variance measures should be available. If there are restrictions on publicly sharing data—e.g. participant privacy or use of data from a third party—those must be specified.

Reviewer #1: No

Reviewer #2: No

**********

4. Is the manuscript presented in an intelligible fashion and written in standard English?

PLOS ONE does not copyedit accepted manuscripts, so the language in submitted articles must be clear, correct, and unambiguous. Any typographical or grammatical errors should be corrected at revision, so please note any specific errors here.

Reviewer #1: Yes

Reviewer #2: Yes

**********

5. Review Comments to the Author

Please use the space provided to explain your answers to the questions above. You may also include additional comments for the author, including concerns about dual publication, research ethics, or publication ethics. (Please upload your review as an attachment if it exceeds 20,000 characters)

Reviewer #1: This study investigated whether temporal binding is enhanced by social perception, specifically by interacting with a face stimulus. Participants performed a time estimation task in active and passive conditions using face and non-social pattern stimuli. Although a trend toward shorter time estimates was observed when participants actively interacted with a face, the results did not provide reliable evidence for temporal binding or social hyperbinding driven by face perception alone.

Overall, the study is well conducted and clearly fits the aims and scope of the journal (in this case, publishing null results). I have only a few minor comments that I believe could help to further improve the clarity and interpretation of the manuscript.

One important limitation concerns the low ecological validity of the stimuli. Although it is well established that highly schematic faces can elicit social attentional processes comparable to those triggered by real faces - please refers to a recent evidence; (Dalmaso et al., 2025) - there remains some uncertainty about how participants actually perceived the different stimuli in the present study. In this respect, the absence of a manipulation check represents a limitation, as such a measure could have helped to assess whether the face stimuli were indeed perceived as socially meaningful and distinct from the pattern stimuli. I would therefore encourage the authors to expand the Discussion by explicitly addressing the issue of ecological validity and stimulus perception, and by considering how this aspect may have contributed to the null results.

I would appreciate some clarification regarding the power analysis. The sample size seems to have been determined using GPower based on a paired-samples t-test effect size, while the data were analysed using linear mixed-effects models. I would therefore welcome clarification on how this power calculation maps onto the mixed-model framework and the specific effects of interest (e.g., interactions), and whether it provides an adequate estimate of statistical power for the reported analyses.

Lastly, I was unclear about the reasons for not sharing the data publicly. Given that the study appears to involve standard behavioural measures (time estimates, experimental conditions, anonymous participant IDs), it is not obvious which privacy concerns would prevent the release of a fully de-identified dataset. I would encourage the authors to clarify this point and, if possible, to consider making the data publicly available in line with open science and replication practices (this is particularly relevant for a journal like Plos One).

Reference

Dalmaso, M., Galfano, G., Baratella, A., & Castelli, L. (2025). A direct comparison of gaze-mediated orienting elicited by schematic and real human faces. Acta Psychologica, 255, 104934. https://doi.org/10.1016/j.actpsy.2025.104934

Reviewer #2: In their study, the authors attempt to replicate findings from previous experiments, where they found a temporal hyperbinding of actions and social effects. In particular, participants reported shorter time intervals between actions and effects they believed to represent another humans behaviour and were represented on a face like stimulus as compared to a geometrical pattern that was not related to another humans behaviour. In the current study they contrasted the two stimuli without a social cover story and found no significant temporal binding at all.

I commend the authors for transparently reporting their null results! Exploring the specific mechanisms and boundary conditions of temporal binding is, I believe, a very current issue which is why these results are very much worth publishing.

However, the lack of raw data and without any (reported) exploratory tests, it is difficult to understand the reason, why the previously found temporal binding effect was absent here.

Major Point 1:

If I understand correctly, the face condition in the current study is identical to the one used in Experiment 1 of Vogel et al. (2021). The only change seems to have been made to the pattern condition, where the movement was adapted to more closely match the movement of the face stimulus. The conditions in Experiment 1 of your study Vogel et al. (2022) are fully identical with the only exception that both original experiments include a cover story, which relates the eye movements to a real person. Despite the face conditions being “on the face of it” identical in all sets, the original studies reported clear temporal binding (TB), whereas the present data do not.

Several interpretations of this come to mind which probably each warrant a separate response. Nevertheless, they all pertain to the relationship between this data and the one from your original study, and may also, especially if I misunderstood or overlooked something, be answered together:

Major 1A: The sample sizes differ. The earlier experiments had a smaller sample (of neurotypical participants), than the current experiment. Although temporal binding often shows a robust mean effect, interindividual differences are notoriously high. It would therefore be informative to examine whether the effect survives a pooled analysis of the three samples. Since this paper is to be published as a stand-alone, I think it would be helpful to include the results you are trying to replicate in one of the graphs. Right now, I had to circle back to your original studies to really grasp the reasoning and the key differences. As figures are quite different between the three manuscripts, a unified depiction would help me (and presumably others) a lot.

Major 1B: The analysis strategy also differed between studies. In your 2021 study, ANOVAs were conducted, whereas the 2022 and the present study uses a mixed model with Subject ID as a random factor. I wonder what motivated you to switch from ANOVAs to mixed models. Honestly, I do not assume that an ANOVA would have given you any different results here. Nevertheless, a direct comparison of both analyses on both datasets would be interesting. As far as I understand, you did do a t-test on the specific contrast already but never reported it.

Major 1C: Modifying the movement of the pattern stimulus may have influenced the overall interpretation of the stimuli, including the perception of the face stimulus. In my opinion, the pattern stimulus has more than just a little resemblance to a sideways face. May the critical factor in the earlier experiment have been not the “socialness” of the face stimulus per se, but rather the perceived socialness of the effect (moving eyes vs shifting overall position). If I read your papers correctly, this is not the case in the 2022 study, but still, the face is associated with a real person here and the pattern is not. I wonder what the authors would predict if the reinstated their cover story for the pattern but not the face. Or if they had the whole face move (like the pattern in the 2021 study) while the pattern only moved the black dots.

I understand completely, that the main problem was a lack of temporal binding altogether. Unless it was pure chance, I still feel like there is much potential here to disentangle the influencing factors and possibly find the relevant conditions that diminish temporal binding.

Major Point 2:

I encourage the authors to report the mean effect for agency, even if (or especially because) it is not statistically significant. It would also be helpful to know whether there is significant temporal binding in the social condition alone. Once again, I am convinced you must have done this test in the “other t-tests that were not significant”, but please share them with the readership.

More generally, I recommend reporting the mean effects and effect sizes for all primary comparisons, along with the results of all follow-up t-tests - simply all tests that you performed. To keep the manuscript slim, you could consider including a comprehensive table of all tests and results in an online repository.

Major Point 3:

Speaking of online repositories, I am not persuaded by the authors’ justification for not sharing their data openly. It sounds very much like a standard phrase as there are no patients included in this study. I strongly urge the authors to share their (anonymized) raw data and analysis scripts on a platform such as the OSF. Open sharing reduces barriers to replication, reanalysis, and meta-analyses. Providing data on demand has multiple disadvantages, as authors can become uncontactable, and data may be lost on local machines. While open platforms are not perfect, they provide an additional layer of security and accessibility for valuable datasets. Please seriously consider this and if it’s not an option for you, please provide a more specific explanation why not for this data set.

In any case, I would request the authors to share the anonymized raw data and analysis script of this experiment as well as the previous studies (at least Experiment 1 from both) with me and all other reviewers for the next round of reviews.

Minor Points

• The statement that temporal binding is strongest under “high temporal predictability and low cognitive load” (ll. 244-245) and may be weakened or masked when task complexity increases, reads as somewhat contrived. While the claim may be correct, it is not self-evident. Please provide supporting citations.

• Additionally, the claim that social information “may draw attention away from temporal processing, leading to compressed duration estimates” (ll.265-266) seems quite at odds with the earlier argument that temporal binding is strongest under low cognitive load.

• The statement that “social stimulus material may not strengthen temporal binding, but may in fact weaken or disrupt it under certain conditions” (ll. 249-250) does not convince me as currently argued. In your experiment, the difference between passive and active trials was smaller, descriptively reversed even, in non-social trials. Additionally, the social connotation in your original experiments was much stronger. Would it not make more sense to consider a possible limitation or flaw in the current experimental design rather than concluding that social stimuli weaken temporal binding?

• The argument beginning with “Given these null results, we urge caution…” at l. 258 - 261 is a bit repetitive.

• The manuscript contains frequent use of “e.g.” and “i.e.”, particularly in the introduction. At least according to APA standard, these should only be used in parentheses. Personally, I find this rule sensible, as these abbreviations disrupt the reading flow so to speak. Of course this is just for your consideration.

Overall, it is an interesting manuscript and especially the reporting of null results aids the current discourse. Still, the discussion is quite ambiguous, as the authors seemingly (and understandably) didn’t really know what to make of the surprising lack of temporal binding. I would be interested in a few more substantiated ideas or exploratory analyses on this, for example in relation to the questions posed in Major Point 1.

I am very much looking forward to reading the authors thoughts on this.

Best wishes!

Annika Klaffehn

**********

6. PLOS authors have the option to publish the peer review history of their article (what does this mean?). If published, this will include your full peer review and any attached files.

If you choose “no”, your identity will remain anonymous but your review may still be made public.

**Do you want your identity to be public for this peer review?**  For information about this choice, including consent withdrawal, please see our Privacy Policy.

Reviewer #1: No

Reviewer #2: **Yes:** Annika L. Klaffehn

**********

[NOTE: If reviewer comments were submitted as an attachment file, they will be attached to this email and accessible via the submission site. Please log into your account, locate the manuscript record, and check for the action link "View Attachments". If this link does not appear, there are no attachment files.]

To ensure your figures meet our technical requirements, please review our figure guidelines: https://journals.plos.org/plosone/s/figures

You may also use PLOS’s free figure tool, NAAS, to help you prepare publication quality figures: https://journals.plos.org/plosone/s/figures#loc-tools-for-figure-preparation.

NAAS will assess whether your figures meet our technical requirements by comparing each figure against our figure specifications.

# Author comment: Author response to Decision Letter 1

**DOI:** 10.1371/journal.pone.0352510.r002

13 Apr 2026

Reviewer Comments to the Author — Point by Point Reply:

Reviewer #1: This study investigated whether temporal binding is enhanced by social perception, specifically by interacting with a face stimulus. Participants performed a time estimation task in active and passive conditions using face and non-social pattern stimuli. Although a trend toward shorter time estimates was observed when participants actively interacted with a face, the results did not provide reliable evidence for temporal binding or social hyperbinding driven by face perception alone.

Overall, the study is well conducted and clearly fits the aims and scope of the journal (in this case, publishing null results). I have only a few minor comments that I believe could help to further improve the clarity and interpretation of the manuscript.

We appreciate the reviewer’s positive feedback.

—————————————————————

One important limitation concerns the low ecological validity of the stimuli. Although it is well established that highly schematic faces can elicit social attentional processes comparable to those triggered by real faces - please refers to a recent evidence; (Dalmaso et al., 2025) - there remains some uncertainty about how participants actually perceived the different stimuli in the present study. In this respect, the absence of a manipulation check represents a limitation, as such a measure could have helped to assess whether the face stimuli were indeed perceived as socially meaningful and distinct from the pattern stimuli. I would therefore encourage the authors to expand the Discussion by explicitly addressing the issue of ecological validity and stimulus perception, and by considering how this aspect may have contributed to the null results.

We thank the reviewer for the close reading of our manuscript and agree that in its current form the limitation brought on by the schematic nature of the stimulus material falls short. We appreciate the addition of the work by Dalmaso et al. (2025), which we had not been aware of to date and now feature in the manuscript. We have amended the discussion section to more thoroughly develop the limitation. The paragraph on page 9 now reads:

“In addition, we did not include a manipulation check to confirm that participants perceived stimuli as intended (i.e., as faces vs. patterns). While schematic faces may evoke attentional processes comparable to those triggered by realistic faces (Dalmaso et al., 2025), we cannot guarantee that such attentional effects transfer to our paradigm. This potential deficit in ecological validity may have negatively influenced effect sizes by reducing emotional intensity or activation of mentalizing or mirroring processes (Vogeley, 2017), subsequently resulting in a perceptual difference between stimuli too subtle to elicit a measurable effect in time estimation. These factors limit the interpretability of any trends and highlight the need for further rigorous paradigms in future research.”

——————————————————

I would appreciate some clarification regarding the power analysis. The sample size seems to have been determined using GPower based on a paired-samples t-test effect size, while the data were analysed using linear mixed-effects models. I would therefore welcome clarification on how this power calculation maps onto the mixed-model framework and the specific effects of interest (e.g., interactions), and whether it provides an adequate estimate of statistical power for the reported analyses.

We thank the reviewer for this important point and appreciate the opportunity to clarify our power analysis.

The a-priori power analysis was conducted in G*Power based on a paired-samples t-test because the only available prior study closely matching our experimental paradigm reported its results using a repeated-measures ANOVA framework (Vogel et al., 2021). The reported effect size was converted to Cohen’s d and used to estimate the required sample size. The selected effect most closely corresponded to the within-subject contrast central to our primary hypothesis. Although our confirmatory analyses were conducted using a linear mixed-effects model, the powered contrast corresponds directly to a fixed effect in the mixed-model framework. We have added the following clarification to the manuscript on page 6:

"We acknowledge that power analyses based on t-tests do not fully account for the additional variance components estimated in LMMs (e.g., random intercepts and slopes). However, given the fully within-subject design and balanced structure, and because the effect size was derived from a comparable paradigm (Vogel et al., 2021), we consider this approach a reasonable and conservative approximation at the planning stage. Importantly, the final sample size exceeded the minimum estimated by the a-priori power analysis.”

—————————————————————————-

Lastly, I was unclear about the reasons for not sharing the data publicly. Given that the study appears to involve standard behavioural measures (time estimates, experimental conditions, anonymous participant IDs), it is not obvious which privacy concerns would prevent the release of a fully de-identified dataset. I would encourage the authors to clarify this point and, if possible, to consider making the data publicly available in line with open science and replication practices (this is particularly relevant for a journal like Plos One).

We wholeheartedly agree with the reviewer. After consultation with both the approving ethics committee, our data security officer, and the journal editor we are now able to publish the data at our discretion.

The data are available at: osf.io/vpyd9

Reviewer #2: In their study, the authors attempt to replicate findings from previous experiments, where they found a temporal hyperbinding of actions and social effects. In particular, participants reported shorter time intervals between actions and effects they believed to represent another human’s behaviour and were represented on a face like stimulus as compared to a geometrical pattern that was not related to another human’s behaviour. In the current study they contrasted the two stimuli without a social cover story and found no significant temporal binding at all.

I commend the authors for transparently reporting their null results! Exploring the specific mechanisms and boundary conditions of temporal binding is, I believe, a very current issue which is why these results are very much worth publishing.

However, the lack of raw data and without any (reported) exploratory tests, it is difficult to understand the reason, why the previously found temporal binding effect was absent here.

We thank the reviewer for their appreciation of our results and fully agree with the stated relevance of boundary conditions to temporal binding (TB) and psychological effects in general. In the following, we will address the important issues the reviewer raises.

—————————————

Major Point 1:

If I understand correctly, the face condition in the current study is identical to the one used in Experiment 1 of Vogel et al. (2021). The only change seems to have been made to the pattern condition, where the movement was adapted to more closely match the movement of the face stimulus. The conditions in Experiment 1 of your study Vogel et al. (2022) are fully identical with the only exception that both original experiments include a cover story, which relates the eye movements to a real person. Despite the face conditions being “on the face of it” identical in all sets, the original studies reported clear temporal binding (TB), whereas the present data do not.

We appreciate the reviewer’s appreciation of our work. Contrary to the reviewer’s understanding, we must point out that the experiments referred to in this comment were identical, but differ from the experiment described in this study. Both the first experiments in Vogel et al., 2021 and 2022 entailed a combination of a face stimulus and a cover story, thereby confounding these two aspects of sociality. In contrast, the current study attempts to clarify the true nature of the influence of face/gaze perception on TB. This confusion underscores the reviewer’s following points which we addressed in a new table 1 explained and depicted below. Table 1 includes a column (named “Effect of Interest”) dedicated at stimulus/cover story combinations in these studies.

—————————————

Several interpretations of this come to mind which probably each warrant a separate response. Nevertheless, they all pertain to the relationship between this data and the one from your original study, and may also, especially if I misunderstood or overlooked something, be answered together:

Major 1A: The sample sizes differ. The earlier experiments had a smaller sample (of neurotypical participants), than the current experiment. Although temporal binding often shows a robust mean effect, interindividual differences are notoriously high. It would therefore be informative to examine whether the effect survives a pooled analysis of the three samples. Since this paper is to be published as a stand-alone, I think it would be helpful to include the results you are trying to replicate in one of the graphs. Right now, I had to circle back to your original studies to really grasp the reasoning and the key differences. As figures are quite different between the three manuscripts, a unified depiction would help me (and presumably others) a lot.

We thank the reviewer for this thoughtful and constructive suggestions. We agree that a clearer, unified depiction of the previously reported effects improves transparency and makes the manuscript more self-contained.

To address this point, we have now added Table 1, which summarizes all relevant experiments from Vogel et al. (2021, 2022) alongside the present study. The table reports the specific effect of interest in each paradigm (i.e., Agency × Partner, Agency × Story, Agency × Stimulus), the corresponding mean temporal binding difference, and the original inferential statistics. For the 2022 study, we additionally report group-specific (TD and ASD) effect magnitudes derived from the published mixed-model coefficients, as well as the formal test of group moderation.

This unified presentation will allow readers to directly compare effects across paradigms and samples without having to consult the earlier manuscripts. It includes the mean social hyperbinding effect. The magnitude observed in the present study (27.31ms) falls within the previously reported range, albeit not reaching statistical significance.

Regarding the suggestion of a pooled analysis, the prior studies differ in design, rendering a direct pooled model across all datasets statistically inappropriate for a replication study. We therefore opted for a structured comparative summary of effect magnitudes and statistical tests across experiments, which we believe more accurately reflects the replication logic.

We hope that this addition improves clarity and addresses the reviewer’s concern about robustness and variability. Please see the added Table 1 in the attached file.

————————————————————-

Major 1B: The analysis strategy also differed between studies. In your 2021 study, ANOVAs were conducted, whereas the 2022 and the present study uses a mixed model with Subject ID as a random factor. I wonder what motivated you to switch from ANOVAs to mixed models. Honestly, I do not assume that an ANOVA would have given you any different results here. Nevertheless, a direct comparison of both analyses on both datasets would be interesting. As far as I understand, you did do a t-test on the specific contrast already but never reported it.

We thank the reviewer for this thoughtful comment. The shift from repeated-measures ANOVA (2021 study) to linear mixed-effects models (2022 and present study) reflects an evolution in our analytic approach rather than a conceptual change. Mixed-effects models allow us to analyze trial-level data without aggregation, appropriately model subject-level variability, and handle potential imbalance or missing data more flexibly. For these reasons, they are now generally recommended for within-subject designs of this kind. However, and as mentioned in your introduction of Major 1, we believe point Major 1B to be complementary to Major 1A. We hence list Test statistics results in Table 1.

————————————————-

Major 1C: Modifying the movement of the pattern stimulus may have influenced the overall interpretation of the stimuli, including the perception of the face stimulus. In my opinion, the pattern stimulus has more than just a little resemblance to a sideways face. May the critical factor in the earlier experiment have been not the “socialness” of the face stimulus per se, but rather the perceived socialness of the effect (moving eyes vs shifting overall position). If I read your papers correctly, this is not the case in the 2022 study, but still, the face is associated with a real person here and the pattern is not. I wonder what the authors would predict if the reinstated their cover story for the pattern but not the face. Or if they had the whole face move (like the pattern in the 2021 study) while the pattern only moved the black dots.

I understand completely that the main problem was a lack of temporal binding altogether. Unless it was pure chance, I still feel like there is much potential here to disentangle the influencing factors and possibly find the relevant conditions that diminish temporal binding.

We thank the reviewer for this highly interesting suggestion concerning further paradigm changes and social binding effects! A comparison between the purely perceptual versus the belief socialness — in other words comparing pattern/story to face/no-story stimuli — would shed further light on the relationship between both aspects of social hyperbinding. Two competing potential results come to mind: a) the null hypothesis with a similar result as seen in the current study; b) increased binding for the pattern/story combination. We strongly favor the latter — particularly in a within subjects design — as in our opinion the previous studies suggest a superiority of or preference for social belief in social temporal binding causation. However, this would require an additional experiment and is beyond the scope of this replication study.

We have added this intriguing idea to the discussion section’s paragraph about future investigations on page 9. We also believe that Table 1 increases readability of this intricate research design. The rewritten paragraph now reads:

“The results of this study emphasize the question about the direct relationship between social perception and social belief. The examination of how interacting with an object of non-human appearance in the belief in a human interactant controlling this object relates to interacting with an object of human appearance knowing that it is not controlled by another person might provide important insight into the relationship between bottom-up and top-down social cognition. Speculatively, as previous work (Vogel et al, 2022), replicated social hyperbinding for social belief without face stimuli (see Table 1), yet our current study did not replicate social hyperbinding for face stimuli without social belief, the introduction of a cover story might elicit increased TB where a social stimulus might not.”

———————————————-

Major Point 2:

I encourage the authors to report the mean effect for agency, even if (or especially because) it is not statistically significant. It would also be helpful to know whether there is significant temporal binding in the social condition alone. Once again, I am convinced you must have done this test in the “other t-tests that were not significant”, but please share them with the readership.

More generally, I recommend reporting the mean effects and effect sizes for all primary comparisons, along with the results of all follow-up t-tests - simply all tests that you performed. To keep the manuscript slim, you could consider including a comprehensive table of all tests and results in an online repository.

We thank the reviewer for this helpful suggestion to improve readability and replicability. We have extended our results section by the req

Attachment Submitted filename: 26-03-18_rebuttal_V1.2.docx

# Peer review: Decision Letter 1

**DOI:** 10.1371/journal.pone.0352510.r003

**Authors:** Elisa Scerrati, Elisa Scerrati

8 May 2026

PONE-D-25-67312R1

Temporal Binding and Social Perception – a Replication Study

PLOS One

Dear Dr.  Vogel,

Thank you for submitting your manuscript to PLOS ONE. After careful consideration, we feel that it has merit but does not fully meet PLOS ONE’s publication criteria as it currently stands. Therefore, we invite you to submit a revised version of the manuscript that addresses the points raised during the review process.

Please submit your revised manuscript by Jun 22 2026 11:59PM. If you will need more time than this to complete your revisions, please reply to this message or contact the journal office at plosone@plos.org. When you're ready to submit your revision, log on to https://www.editorialmanager.com/pone/ and select the 'Submissions Needing Revision' folder to locate your manuscript file.

Please include the following items when submitting your revised manuscript:

- A letter that responds to each point raised by the academic editor and reviewer(s). You should upload this letter as a separate file labeled 'Response to Reviewers'.
- A marked-up copy of your manuscript that highlights changes made to the original version. You should upload this as a separate file labeled 'Revised Manuscript with Track Changes'.
- An unmarked version of your revised paper without tracked changes. You should upload this as a separate file labeled 'Manuscript'.

If you would like to make changes to your financial disclosure, please include your updated statement in your cover letter. Guidelines for resubmitting your figure files are available below the reviewer comments at the end of this letter.

If applicable, we recommend that you deposit your laboratory protocols in protocols.io to enhance the reproducibility of your results. Protocols.io assigns your protocol its own identifier (DOI) so that it can be cited independently in the future. For instructions see: https://journals.plos.org/plosone/s/submission-guidelines#loc-laboratory-protocols. Additionally, PLOS ONE offers an option for publishing peer-reviewed Lab Protocol articles, which describe protocols hosted on protocols.io. Read more information on sharing protocols at https://plos.org/protocols?utm_medium=editorial-email&utm_source=authorletters&utm_campaign=protocols.

As the corresponding author, your ORCID iD is verified in the submission system and will appear in the published article. PLOS supports the use of ORCID, and we encourage all coauthors to register for an ORCID iD and use it as well. Please encourage your coauthors to verify their ORCID iD within the submission system before final acceptance, as unverified ORCID iDs will not appear in the published article. *Only*  the individual author can complete the verification step; PLOS staff *cannot*  verify ORCID iDs on behalf of authors.

We look forward to receiving your revised manuscript.

Kind regards,

Elisa Scerrati

Academic Editor

PLOS One

Journal Requirements:

1. If the reviewer comments include a recommendation to cite specific previously published works, please review and evaluate these publications to determine whether they are relevant and should be cited. There is no requirement to cite these works unless the editor has indicated otherwise.

2. Please review your reference list to ensure that it is complete and correct. If you have cited papers that have been retracted, please include the rationale for doing so in the manuscript text, or remove these references and replace them with relevant current references. Any changes to the reference list should be mentioned in the rebuttal letter that accompanies your revised manuscript. If you need to cite a retracted article, indicate the article’s retracted status in the References list and also include a citation and full reference for the retraction notice.

Additional Editor Comments:

While both Reviewers acknowledge the improvements resulting from your reviews, Reviewer 2 point out some minor linguistic issues and a more serious concern about power analysis, which I strongly encourage you to consider.

[Note: HTML markup is below. Please do not edit.]

Reviewer's Responses to Questions

**Comments to the Author**

1. If the authors have adequately addressed your comments raised in a previous round of review and you feel that this manuscript is now acceptable for publication, you may indicate that here to bypass the “Comments to the Author” section, enter your conflict of interest statement in the “Confidential to Editor” section, and submit your "Accept" recommendation.

Reviewer #1: All comments have been addressed

Reviewer #2: (No Response)

**********

2. Is the manuscript technically sound, and do the data support the conclusions?

The manuscript must describe a technically sound piece of scientific research with data that supports the conclusions. Experiments must have been conducted rigorously, with appropriate controls, replication, and sample sizes. The conclusions must be drawn appropriately based on the data presented.

Reviewer #1: Yes

Reviewer #2: Yes

**********

3. Has the statistical analysis been performed appropriately and rigorously?

Reviewer #1: Yes

Reviewer #2: Yes

**********

4. Have the authors made all data underlying the findings in their manuscript fully available?

The PLOS Data policy requires authors to make all data underlying the findings described in their manuscript fully available without restriction, with rare exception (please refer to the Data Availability Statement in the manuscript PDF file). The data should be provided as part of the manuscript or its supporting information, or deposited to a public repository. For example, in addition to summary statistics, the data points behind means, medians and variance measures should be available. If there are restrictions on publicly sharing data—e.g. participant privacy or use of data from a third party—those must be specified.

Reviewer #1: Yes

Reviewer #2: Yes

**********

5. Is the manuscript presented in an intelligible fashion and written in standard English?

PLOS ONE does not copyedit accepted manuscripts, so the language in submitted articles must be clear, correct, and unambiguous. Any typographical or grammatical errors should be corrected at revision, so please note any specific errors here.

Reviewer #1: Yes

Reviewer #2: Yes

**********

6. Review Comments to the Author

Please use the space provided to explain your answers to the questions above. You may also include additional comments for the author, including concerns about dual publication, research ethics, or publication ethics. (Please upload your review as an attachment if it exceeds 20,000 characters)

Reviewer #1: I am happy with this new version. I can therefore suggest the publication of this paper in the current form.

Reviewer #2: Dear Authors

Thank you for uploading the raw data. While I admit I was a bit disappointed that you did not share my enthusiasm for the exploratory cross-experiment analyses, which I would have found worthwhile despite the differences in cover stories, I do understand the reasoning. In any case, making the full dataset available largely addresses this concern, as interested readers can carry out these analyses themselves. I also appreciate your explanation regarding the discussion and the somewhat loosely connected set of hypotheses. Although the revisions in this section appear modest, in my opinion the text reads more clearly now. In particular, the section summarizing the study’s limitations is very helpful. Thank you as well for including a table with the summary statistics, which, as you put it, makes the article more self-contained. Overall, I believe the article is almost ready for publication. While reading, I only noticed a few minor errors and some slightly awkward phrasings that seem to have been overlooked during internal review. Additionally, I would ask the authors to review their power analysis once more, as I was not totally convinced by the changes they made.

P. 2 “However, while watching another person perform the task [6] or when performing the task with another person [5, 8, 16], TB is shorter than while performing the task alone. “

“TB is shorter” seems very weird to me and left me wondering whether you actually wanted to say that ratings became shorter. I guess the TB effect became smaller or decreased would be clearer.

P.3 “For one, TB has been thought to depend on the Sense of Agency –“

I stumbled a bit over this sentence. I’m not a native speaker so feel free to completely ignore me if I’m wrong, but is “thought” correct here? I would have expected something like “considered”, “believed” or “theorized”.

P. 4 “For half of the trials participants were made to believe they were interacting with another person, while during the other half, they were told to be performing the experiment by themselves.”

“…they were told to be performing the experiment by themselves.” does not seem correct. “…they were told they would be performing the experiment by themselves.”?

p. 5 “resulting in a minimum sample size per group at n = 22.”

What are the groups you speak of here? Why did you not calculate an effect size for within subjects analyses, if that was your plan all along? It reads as if it was simply an error made at the time. If that is the case, it is understandable but to avoid confusion you could also simply say that you based your sample size on the effect size from Vogel et al. 2019 (and report this original effect size). The calculated required sample was 22 participants, then add your explanation that you deliberately overshot this estimate in order to account for changes. Crucially, you could skip the lengthy and slightly awkward explanation of why ANOVA based power analyses are more or less appropriate and just instead run a post hoc simulation based power estimation for the LMM with your final sample size and report how much power you ended up with.

p. 7 “All remaining contrasts were non-significant both before and after correction (active/pattern vs. passive/face: M = 5.56, SE = 14.57, t(39) = 0.382, p = 0.705, pbonf = 1.000; passive/face vs. active/face: M = 24.44, SE =15.32, t(39) = 1.595, p = 0.119, p pbonf = 0.712; passive/pattern vs. active/face: M = 27.14, SE = 16.75, t(39) = 1.620, p = .113, p pbonf = 0.680; passive/pattern vs. active/pattern: M = −2.87, SE = 15.01, t(39) = −0.191, p = 0.849, p pbonf = 1.000; passive/pattern vs. passive/face: M = 2.69, SE = 9.53, t(39) = 0.283, p = 0.779, p pbonf = 1.000).”

I think there are a couple of lonely ps before some pbonfs in this paragraph.

p.8 “as a possible limitation of flaw in our paradigm”

it should be or, not of, right?

That is all, all the best!

Annika Klaffehn

**********

7. PLOS authors have the option to publish the peer review history of their article (what does this mean?). If published, this will include your full peer review and any attached files.

If you choose “no”, your identity will remain anonymous but your review may still be made public.

**Do you want your identity to be public for this peer review?**  For information about this choice, including consent withdrawal, please see our Privacy Policy.

Reviewer #1: No

Reviewer #2: **Yes:** Annika L. Klaffehn

**********

[NOTE: If reviewer comments were submitted as an attachment file, they will be attached to this email and accessible via the submission site. Please log into your account, locate the manuscript record, and check for the action link "View Attachments". If this link does not appear, there are no attachment files.]

To ensure your figures meet our technical requirements, please review our figure guidelines: https://journals.plos.org/plosone/s/figures

You may also use PLOS’s free figure tool, NAAS, to help you prepare publication quality figures: https://journals.plos.org/plosone/s/figures#loc-tools-for-figure-preparation.

NAAS will assess whether your figures meet our technical requirements by comparing each figure against our figure specifications.

# Author comment: Author response to Decision Letter 2

**DOI:** 10.1371/journal.pone.0352510.r004

19 May 2026

Reviewer #2:

Dear Authors

Thank you for uploading the raw data. While I admit I was a bit disappointed that you did not share my enthusiasm for the exploratory cross-experiment analyses, which I would have found worthwhile despite the differences in cover stories, I do understand the reasoning. In any case, making the full dataset available largely addresses this concern, as interested readers can carry out these analyses themselves. I also appreciate your explanation regarding the discussion and the somewhat loosely connected set of hypotheses. Although the revisions in this section appear modest, in my opinion the text reads more clearly now. In particular, the section summarizing the study’s limitations is very helpful. Thank you as well for including a table with the summary statistics, which, as you put it, makes the article more self-contained. Overall, I believe the article is almost ready for publication. While reading, I only noticed a few minor errors and some slightly awkward phrasings that seem to have been overlooked during internal review. Additionally, I would ask the authors to review their power analysis once more, as I was not totally convinced by the changes they made.

We thank the reviewer for the positive feedback. We too believe the changes recommended in the last round of reviews have greatly improved the manuscript, particularly concerning clarity. We apologize for any errors that persisted after the first review and have now taken great care of correcting them.

P. 2 “However, while watching another person perform the task [6] or when performing the task with another person [5, 8, 16], TB is shorter than while performing the task alone. “

“TB is shorter” seems very weird to me and left me wondering whether you actually wanted to say that ratings became shorter. I guess the TB effect became smaller or decreased would be clearer.

We agree with the reviewer that our choice of phrasing was confusing. We have changed the sentence to:

“However, while watching another person perform the task [6] or when performing the task with another person [5, 8, 16], TB is smaller than while performing the task alone.”

P.3 “For one, TB has been thought to depend on the Sense of Agency –“

I stumbled a bit over this sentence. I’m not a native speaker so feel free to completely ignore me if I’m wrong, but is “thought” correct here? I would have expected something like “considered”, “believed” or “theorized”.

We have changed the sentence to:

“For one, TB has been theorized to depend on the Sense of Agency – […]”

P. 4 “For half of the trials participants were made to believe they were interacting with another person, while during the other half, they were told to be performing the experiment by themselves.”

“…they were told to be performing the experiment by themselves.” does not seem correct. “…they were told they would be performing the experiment by themselves.”?

We thank the reviewer for pointing this out. We agree that the original phrasing was unclear and have revised the sentence to:

“For half of the trials participants were made to believe they were interacting with another person, while during the other half, they were told they would perform the experiment by themselves.”

p. 5 “resulting in a minimum sample size per group at n = 22.”

What are the groups you speak of here? Why did you not calculate an effect size for within subjects analyses, if that was your plan all along? It reads as if it was simply an error made at the time. If that is the case, it is understandable but to avoid confusion you could also simply say that you based your sample size on the effect size from Vogel et al. 2019 (and report this original effect size). The calculated required sample was 22 participants, then add your explanation that you deliberately overshot this estimate in order to account for changes. Crucially, you could skip the lengthy and slightly awkward explanation of why ANOVA based power analyses are more or less appropriate and just instead run a post hoc simulation based power estimation for the LMM with your final sample size and report how much power you ended up with.

We thank the reviewer for this helpful suggestion. We agree that the original wording regarding “groups” was imprecise in the context of a fully within-subject design and that the previous justification of the a-priori power analysis was unnecessarily extensive.

In the revised manuscript, we therefore simplified the description of the initial sample size estimation. As originally intended, we now state that the recruitment target was based on the effect size reported by Vogel et al. (2021) using a paired-samples framework appropriate for the original planning stage, and that we deliberately exceeded this estimate to account for modifications to the paradigm and analysis strategy. The revised section reads:

“Based on the effect sizes reported by Vogel et al. (2021) [9], we initially conducted an a priori power analysis in G*Power [31] for paired-samples comparisons with ⍺ = 0.05, desired power = 0.85, Cohen’s dz = 0.68, yielding a minimum target sample size of 22 participants. To account for modifications to the original paradigm and the use of linear mixed-effects modelling, we deliberately exceeded this estimate and recruited 40 participants (16 identifying as male, 24 identifying as female; mean age 28.2, SD 6.85).”

Importantly, following the reviewer’s recommendation, we additionally conducted a post hoc simulation-based power analysis for the final linear mixed-effects model using the observed fixed and random effect structure and the final sample size. We placed this analysis in the Results section and now report the achieved power for the relevant model effects. Because the Agency × Stimulus interaction constituted the primary effect of theoretical interest with regard to social hyperbinding, we specifically highlight that the achieved power for this interaction was moderate (1-β = .48). The section added at the end of the results section reads:

“To evaluate the sensitivity of the present design, we conducted a post-hoc simulation-based power analysis using jamovi’s pamlj module [40] for the final linear mixed-effects model using the observed fixed and random effect structure and the final sample size (N = 40; 8000 observations total). The simulation indicated excellent power for detecting the main effect of interval (1-β = 1.00). However, the power for the main effects of agency and stimulus was low (1-β = 0.14) to moderate (1-β = .51), respectively. The power for the stimulus × interval interaction (1-β = 0.74) and the agency × stimulus interaction – the primary effect of theoretical interest regarding social hyperbinding – was moderate (1-β = 0.48), while the power for the agency × interval interaction was low (1-β = .15). These findings suggest that although the present design was sufficiently sensitive to detect large interval-related effects, it had a reduced sensitivity for smaller agency-related interaction effects. Consequently, the absence of statistically significant evidence for social hyperbinding in the present study should be interpreted cautiously, as smaller interaction effects may have remained undetected.”

p. 7 “All remaining contrasts were non-significant both before and after correction (active/pattern vs. passive/face: M = 5.56, SE = 14.57, t(39) = 0.382, p = 0.705, pbonf = 1.000; passive/face vs. active/face: M = 24.44, SE =15.32, t(39) = 1.595, p = 0.119, p pbonf = 0.712; passive/pattern vs. active/face: M = 27.14, SE = 16.75, t(39) = 1.620, p = .113, p pbonf = 0.680; passive/pattern vs. active/pattern: M = −2.87, SE = 15.01, t(39) = −0.191, p = 0.849, p pbonf = 1.000; passive/pattern vs. passive/face: M = 2.69, SE = 9.53, t(39) = 0.283, p = 0.779, p pbonf = 1.000).”

I think there are a couple of lonely ps before some pbonfs in this paragraph.

The lonely ps have been removed.

p.8 “as a possible limitation of flaw in our paradigm”

it should be or, not of, right?

The typeo has been corrected. Thank you for pointing this out.

Attachment Submitted filename: 26-05-15_V5.2-rebuttal.docx

# Peer review: Decision Letter 2

**DOI:** 10.1371/journal.pone.0352510.r005

**Authors:** Elisa Scerrati, Elisa Scerrati, Elisa Scerrati

11 Jun 2026

Temporal Binding and Social Perception – a Replication Study

PONE-D-25-67312R2

Dear Dr. Vogel,

We’re pleased to inform you that your manuscript has been judged scientifically suitable for publication and will be formally accepted for publication once it meets all outstanding technical requirements.

Within one week, you’ll receive an e-mail detailing the required amendments. When these have been addressed, you’ll receive a formal acceptance letter and your manuscript will be scheduled for publication.

An invoice will be generated when your article is formally accepted. Please note, if your institution has a publishing partnership with PLOS and your article meets the relevant criteria, all or part of your publication costs will be covered. Please make sure your user information is up-to-date by logging into Editorial Manager at Editorial Manager® and clicking the ‘Update My Information' link at the top of the page. For questions related to billing, please contact billing support.

If your institution or institutions have a press office, please notify them about your upcoming paper to help maximize its impact. If they’ll be preparing press materials, please inform our press team as soon as possible -- no later than 48 hours after receiving the formal acceptance. Your manuscript will remain under strict press embargo until 2 pm Eastern Time on the date of publication. For more information, please contact onepress@plos.org.

Kind regards,

Elisa Scerrati

Academic Editor

PLOS One

Additional Editor Comments (optional):

Reviewers' comments:

Reviewer's Responses to Questions

**Comments to the Author**

1. If the authors have adequately addressed your comments raised in a previous round of review and you feel that this manuscript is now acceptable for publication, you may indicate that here to bypass the “Comments to the Author” section, enter your conflict of interest statement in the “Confidential to Editor” section, and submit your "Accept" recommendation.

Reviewer #2: All comments have been addressed

**********

2. Is the manuscript technically sound, and do the data support the conclusions?

The manuscript must describe a technically sound piece of scientific research with data that supports the conclusions. Experiments must have been conducted rigorously, with appropriate controls, replication, and sample sizes. The conclusions must be drawn appropriately based on the data presented.

Reviewer #2: Yes

**********

3. Has the statistical analysis been performed appropriately and rigorously?

Reviewer #2: Yes

**********

4. Have the authors made all data underlying the findings in their manuscript fully available?

The PLOS Data policy requires authors to make all data underlying the findings described in their manuscript fully available without restriction, with rare exception (please refer to the Data Availability Statement in the manuscript PDF file). The data should be provided as part of the manuscript or its supporting information, or deposited to a public repository. For example, in addition to summary statistics, the data points behind means, medians and variance measures should be available. If there are restrictions on publicly sharing data—e.g. participant privacy or use of data from a third party—those must be specified.

Reviewer #2: Yes

**********

5. Is the manuscript presented in an intelligible fashion and written in standard English?

PLOS ONE does not copyedit accepted manuscripts, so the language in submitted articles must be clear, correct, and unambiguous. Any typographical or grammatical errors should be corrected at revision, so please note any specific errors here.

Reviewer #2: Yes

**********

6. Review Comments to the Author

Please use the space provided to explain your answers to the questions above. You may also include additional comments for the author, including concerns about dual publication, research ethics, or publication ethics. (Please upload your review as an attachment if it exceeds 20,000 characters)

Reviewer #2: Congratulations on your interesting paper. All my concerns have been addressed, and I believe it is ready for publication.

**********

7. PLOS authors have the option to publish the peer review history of their article (what does this mean?). If published, this will include your full peer review and any attached files.

If you choose “no”, your identity will remain anonymous but your review may still be made public.

**Do you want your identity to be public for this peer review?**  For information about this choice, including consent withdrawal, please see our Privacy Policy.

Reviewer #2: **Yes:** Annika L. Klaffehn

**********

# Editor report: Acceptance letter

**DOI:** 10.1371/journal.pone.0352510.r006

**Authors:** Elisa Scerrati, Elisa Scerrati, Elisa Scerrati

PONE-D-25-67312R2

PLOS One

Dear Dr. Vogel,

I'm pleased to inform you that your manuscript has been deemed suitable for publication in PLOS One. Congratulations! Your manuscript is now being handed over to our production team.

At this stage, our production department will prepare your paper for publication. This includes ensuring the following:

* All references, tables, and figures are properly cited

* All relevant supporting information is included in the manuscript submission,

* There are no issues that prevent the paper from being properly typeset

You will receive further instructions from the production team, including instructions on how to review your proof when it is ready. Please keep in mind that we are working through a large volume of accepted articles, so please give us a few days to review your paper and let you know the next and final steps.

Lastly, if your institution or institutions have a press office, please let them know about your upcoming paper now to help maximize its impact. If they'll be preparing press materials, please inform our press team within the next 48 hours. Your manuscript will remain under strict press embargo until 2 pm Eastern Time on the date of publication. For more information, please contact onepress@plos.org.

You will receive an invoice from PLOS for your publication fee after your manuscript has reached the completed accept phase. If you receive an email requesting payment before acceptance or for any other service, this may be a phishing scheme. Learn how to identify phishing emails and protect your accounts at https://explore.plos.org/phishing.

If we can help with anything else, please email us at customercare@plos.org.

Thank you for submitting your work to PLOS ONE and supporting open access.

Kind regards,

PLOS ONE Editorial Office Staff

on behalf of

Dr. Elisa Scerrati

Academic Editor

PLOS One
